# The antimicrobial protein S100A12 identified as a potential autoantigen in a subgroup of atopic dermatitis patients

**DOI:** 10.1186/s13601-019-0240-4

**Published:** 2019-01-31

**Authors:** Maria Mikus, Catharina Johansson, Nathalie Acevedo, Peter Nilsson, Annika Scheynius

**Affiliations:** 1 Affinity Proteomics, Department of Protein Science, SciLifeLab, KTH Royal Institute of Technology, Stockholm, Sweden; 2 Department of Clinical Science and Education, Karolinska Institutet, and Sachs’ Children and Youth Hospital, Södersjukhuset, Stockholm, Sweden; 3 Institute for Immunological Research, University of Cartagena, Cartagena, Colombia; 4 Clinical Genomics, SciLifeLab, Stockholm, Sweden

**Keywords:** Affinity proteomics, Antimicrobial protein, Atopic dermatitis/eczema, Autoantibody profiling, Autoantigen, Autoimmunity, Co-morbidity, IgG, Protein microarrays, Suspension bead array

## Abstract

**Background:**

Atopic dermatitis (AD) is a complex heterogeneous chronic inflammatory skin disease. Specific IgE antibodies against autoantigens have been observed in a subgroup of AD patients, however, little is known about IgG-auto-reactivity in AD. To investigate the presence of autoreactive IgG antibodies, we performed autoantibody profiling of IgG in patients with AD of different severities and in healthy controls (HC).

**Methods:**

First, we performed an untargeted screening in plasma samples from 40 severe AD (sAD) patients and 40 HC towards 1152 protein fragments on planar antigen microarrays. Next, based on the findings and addition of more fragments, a targeted antigen suspension bead array was designed to profile a cohort of 50 sAD patients, 123 patients with moderate AD (mAD), and 84 HC against 148 protein fragments representing 96 unique proteins.

**Results:**

Forty-nine percent of the AD patients showed increased IgG-reactivity to any of the four antigens representing keratin associated protein 17-1 (KRTAP17-1), heat shock protein family A (Hsp70) member 4 (HSPA4), S100 calcium binding proteins A12 (S100A12), and Z (S100Z). The reactivity was more frequent in the sAD patients (66%) than in those with mAD (41%), whereas only present in 25% of the HC. IgG-reactivity to S100A12, a protein including an antimicrobial peptide, was only observed in AD patients (13/173).

**Conclusions:**

Autoantibody profiling of IgG-reactivity using microarray technology revealed an autoantibody-based subgroup in patients with AD. The four identified autoantigens and especially S100A12 could, if characterized further, increase the understanding of different pathogenic mechanisms behind AD and thereby enable better treatment.

**Electronic supplementary material:**

The online version of this article (10.1186/s13601-019-0240-4) contains supplementary material, which is available to authorized users.

## Background

Atopic dermatitis (AD, also designated atopic eczema) is a complex heterogeneous chronic inflammatory skin disease [1] with 15–30% of children and 2–10% of adults being afflicted [2]. Defects in the skin barrier combined with inappropriate immune responses are considered central in the pathogenesis of AD with contribution of both genetic/epigenetic and environmental factors including microbial agents [3–5]. Today there is an increased awareness that AD not only is associated with other atopic conditions but also has comorbidities with other diseases such as autoimmune and cardiovascular diseases suggesting that AD is a systemic disorder [6]. The different mechanisms behind the development of allergy related symptoms and the presence of comorbidities highlight the need to characterize the biological differences behind the complex clinical heterogeneity of AD to identify the proper endotypes to match novel specific biological treatments [7–9].

AD has been classified into an extrinsic or an intrinsic type according to the presence or absence of allergen-specific IgE antibodies [10]. The extrinsic AD phenotype is frequently characterized by coexistence of several IgE-mediated symptoms such as eczema, rhinitis and asthma, and polysensitization indicating that type 2 signaling pathways in the immune system participate in the disease development [7]. The other subgroup, consisting of around 20% of the AD patients, includes those with a clinical picture of AD but who lack detectable IgE antibodies to known food or aeroallergens and they usually have normal total serum IgE levels [10]. At present, one may therefore define this group as suffering from non-IgE-associated forms of AD [11], but it is also likely that the corresponding allergens triggering the skin inflammation in this group are not yet defined [12].

Still another subgroup of patients with AD has, in addition to sensitization against exogenous allergens, an IgE-mediated reactivity against autoantigens like epidermal proteins [13, 14]. These patients usually have a more severe form of AD including high levels of circulating IgE [15]. In addition, to add to the complexity, cross-reactivity between fungal allergens and the human homologues manganese superoxide dismutase [16] and thioredoxin [17] has been observed in AD patients sensitized to the skin commensal yeast *Malassezia sympodialis* [18, 19]. Autoimmunity is usually connected with increased levels of IgG to self-structures. The term autoallergy refers to autoimmunity accompanying an atopic disease with antigen-specific IgE raised against self-proteins [20]. Although IgG-reactivity (and IgE-reactivity) has been detected towards dense fine speckled autoantigen of 70 kD (DFS70) in AD patients [21, 22], little is in general known about IgG-auto-reactivity in AD.

In this study we therefore took the approach to perform a meticulous analysis of the IgG-reactivity profiles towards a broad panel of human protein fragments in AD patients compared with healthy controls (HC) using in-house developed protein microarrays [23–27]. The aim was to obtain information if and how IgG-reactivity may be linked with different clinical phenotypes of AD including co-morbidity with respiratory symptoms. Characterization of autoreactive IgG antibodies and identification of autoantigens could increase the understanding of different pathogenic mechanisms behind AD and thereby enable specific prevention and treatments.

## Methods

### Study population and IgE serology

Adult AD patients and HC were recruited in Stockholm, Sweden, during September until May to avoid the summer season, as described previously [15]. Inclusion criteria for the AD patients were diagnosis, set by a dermatologist, according to the UK working party criteria [28]. Exclusion criteria were skin diseases other than AD, autoimmune diseases, immune deficiencies, malignant diseases, pregnancy or lactation, immunosuppressive treatment and an age below 18 or above 65 years. Use of systemic glucocorticoids or systemic antifungal treatment was not allowed for 2 months before the investigation. The severity of the eczema was assessed using SCORing Atopic Dermatitis (SCORAD) [29] where after patients with moderate AD (mAD) or severe AD (sAD) not only restricted to the hands were subjected to the study. The history of patient reported respiratory allergic symptoms was documented as co-morbidity. The HC had no clinical symptoms or history of allergy or skin diseases.

Total IgE and allergen-specific IgE to any of 11 common aeroallergen sources (dog, cat, horse, birch, timothy, mugwort, pellitory, olive tree, *Cladosporium*, *Dermatophagoides pteronyssinus,* and *Dermatophagoides farinae*) included in the mix Phadiatop, were measured in plasma using ImmunoCAP (Thermo Fisher Scientific, Uppsala, Sweden) during 2006–2012 at the Karolinska University Hospital, Stockholm. The reference range for total plasma IgE was 1.6–122 kU/L and for a positive Phadiatop ≥ 0.35 kU/L.

### Study design

**Fig. 1:**
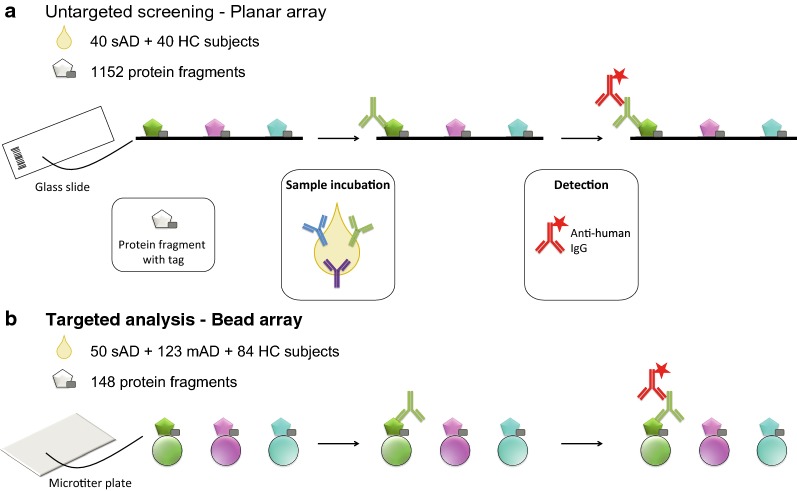
Study design and method overview. **a** In the initial untargeted screening, a subset of 80 subjects was analyzed for IgG-reactivity towards 1152 protein fragments using planar antigen arrays. **b** In the targeted analysis phase, the complete cohort of 257 subjects was profiled on antigen suspension bead arrays including 148 protein fragments selected from the screening as well as from literature (see Additional file 1: Table S1). Planar and bead-based arrays included protein fragments that were either spotted on the array glass slides (**a**) or coupled to the beads (**b**). Antigens were human protein fragments expressed with an N-terminal hexa-histidine albumin binding protein (His6-ABP) tag. Arrays were incubated with plasma and autoantibodies detected using labeled anti-human IgG. *HC* healthy controls, *AD* atopic dermatitis, *mAD* moderate AD, *sAD* severe AD

To profile plasma IgG-reactivity in patients with AD and HC we used two different microarray platforms (see Fig. 1 for an overview of the study design). First, we performed an untargeted screening in 80 subjects using more than 1100 protein fragments on planar antigen microarrays. To verify findings from this initial screening phase and with use of additional fragments (see Additional file 1: Table S1), a targeted antigen suspension bead array was designed to profile the complete cohort of 257 subjects against 148 protein fragments.

### Antigens

The antigens used in this study were human protein fragments produced within the Human Protein Atlas project (www.proteinatlas.org) as described previously [30–32]. Briefly, protein fragments with an average length of 80 amino acids and mostly ranging between 50 and 150 amino acid residues were designed in silico with low homology to other human proteins, cloned and expressed in *Escherichia coli* (*E. coli*) as fusion proteins with an N-terminal hexa-histidine albumin binding protein (His6-ABP) tag, and validated with mass spectrometry subsequent to immobilized metal affinity chromatography purification. Protter v 1.0 (http://wlab.ethz.ch/protter/start/) [33] was used for visualization of protein sequence and region of fragments.

### Planar antigen microarrays

In the screening phase of the study, planar antigen microarrays produced in the routine antibody validation workflow within the Human Protein Atlas project [34] (www.proteinatlas.org) were used to generate autoantibody profiles in plasma for 80 subjects. Each microarray slide consisted of 21 identical subarrays, each subarray containing 384 protein fragments. Three of these array designs comprising in total 1152 protein fragments were selected randomly and used in an untargeted screening. The screening was performed as described previously with minor changes [23], see Supplementary Methods in Additional file 1. Data in the form of median fluorescence intensity (MFI) per array spot were used in further analysis. Cut-off levels for positive IgG-reactivity were set in a sample-specific manner to account for the individual background of each sample. The cut-off was calculated based on the sample median of all 384 protein fragments on each of the subarrays plus 10 or 30 times the median absolute deviation (MAD). For each sample, the signal of individual protein fragments was then compared to the cut-off and positive IgG-reactivity to a particular fragment defined as a fragment signal above the cut-off. The frequency of reactive individuals in sAD and HC were compared and protein fragments selected for further analysis in the targeted screening based on the following criteria: Fisher’s exact test *P* value < 0.05 or ≥ 10% unit difference.

### Antigen suspension bead arrays

A targeted antigen suspension bead array was designed to profile the complete cohort including 257 subjects (Fig. 1). This design was based on results from the initial screening, including the top findings as defined from the above mentioned criteria of statistical significance or frequency of reactivity. Additional protein fragments were included to represent targets from the literature [35–38]. Availability of protein fragments within the Human Protein Atlas (www.proteinatlas.org) resulted in a set of 148 fragments representing 96 proteins (see Additional file 1: Table S1 for a list of all fragments, sequences and the source for inclusion). For 62 of the 96 proteins, each protein was represented by two to four fragments that covered different regions of the protein (exemplified in Additional file 1: Fig S1B and D where two fragments were included for these proteins). The protein fragments were coupled to color-coded carboxylated magnetic beads (MagPlex, Luminex corp., Austin, TX, USA) to create an antigen suspension bead array that was incubated with the samples as previously described [23], see Supplementary Methods for more details. Data in the form of MFI, counted on at least 30 beads per fragment, were compared to sample-specific cut-offs as described for the planar array. For each sample the median MFI across all 148 fragments plus a factor of 10–70 times the MAD were used to create cut-off levels. Again, positive IgG-reactivity was determined by relating the signal of individual protein fragments to the cut-off. Signal intensities were also compared across groups after transformation where the sample median MFI across all fragments was subtracted from the raw MFI and divided by the MAD, thereby creating adjusted MFI values where the signal is expressed in MADs above or below the median. Results are expressed as frequency of reactive individuals (% of subjects with IgG-reactivity above the cut-off level) and as intensity expressed as adjusted MFI.

### Statistical analysis

Data analysis and visualization were performed in R [39]. Fisher’s exact tests were used per protein fragment to compare reactivity frequencies between groups. Wilcoxon rank-sum tests were used per fragment to compare intensity levels (adjusted MFI) between groups. Unadjusted *P* values < 0.05 were here considered significant. Hierarchical clustering was performed on binary data (IgG-reactivity “yes” or “no”).

## Results

### Study population

In total 173 adult AD patients, 18–65 years (median 28 years), and 84 HC, 18–65 years (median 39 years) were included in the study according to the inclusion and exclusion criteria in the Methods. Clinical and demographic characteristics are presented in Table 1. Among the AD patients, 29% were classified as sAD and 71% as mAD. The majority of patients with AD had current or a history of rhinoconjunctivitis and/or asthma (80%). Total IgE levels were elevated (≥ 122 kU/L) in 55% of the AD patients, 70% of patients with sAD and in 49% of patients with mAD. A positive Phadiatop, defined as detectable IgE antibodies to a mixture of 11 common aeroallergens, was found in 76% of the AD patients.

**Table 1:** Characterization of the AD patients and healthy controls

	N	Gender F/M	Age (years)	SCORAD^a^	Rhino-conjunctivitis and/or asthma current or history	Total plasma IgE^b^ ≥ 122 kU/L	Total plasma IgE^b^ (kU/L)	Phadiatop positive^c^
		n (% F)	Median (range)	Median (range)	n (%)	n (%)	Median (range)	n (%)
AD patients	173	99/74 (57)	28 (18–65)	34 (14–70)	139 (80)	95 (55)	160 (2–15,100)	132 (76)
Severe^a^	50	17/33 (34)	29 (18–65)	54 (41–70)	45 (90)	35 (70)	460 (2.5–15,100)	43 (86)
Moderate^a^	123	82/41 (67)	28 (18–63)	31 (14–40)	94 (76)	60 (49)	120 (2–6810)	89 (72)
Healthy controls	84	52/32 (62)	39 (18–65)	NA	0 (0)	0 (0)	19 (2–120)	0 (0)

*AD* atopic dermatitis, *F* female, *M* male, *N* number of individuals, *n* number of positive individuals, *NA* not applicable

^a^Objective SCORAD [29], severe AD defined as SCORAD ≥ 41

^b^ImmunoCAP (Thermo Fisher Scientific), reference range 1.6–122 kU/L

^c^Phadiatop (Thermo Fisher Scientific), plasma IgE-reactivity to any of 11 common aeroallergens, reference range ≥ 0.35 kU/L

### Untargeted screening

In the first screening phase (Fig. 1a), antigens representing keratin associated protein 17-1 (KRTAP17-1) and S100 calcium binding protein Z (S100Z) together with 21 additional antigens showed a difference in the frequency of IgG-reactivity between sAD patients (n = 40) and HC (n = 40) of at least 10% units. Three of these antigens (representing the family with sequence similarity 9 member C, FAM9C; the olfactory receptor family 2 subfamily T member 11, OR2T11; and the S100 calcium binding protein A3, S100A3) had significantly higher reactivity in sAD compared to HC (*P* < 0.05). These in total 23 antigens were selected for further analysis in the targeted analysis phase (listed in Additional file 1: Table S1 as “Screening (*P *< 0.05)” or “Screening (≥ 10% reactivity difference)”). Additional fragments that had not been represented on the planar antigen arrays and that covered other regions of the represented proteins were also included. This resulted in a final selection of 47 protein fragments based on the screening phase (see Additional file 1: Table S1).

### Targeted analysis

**Fig. 2:**
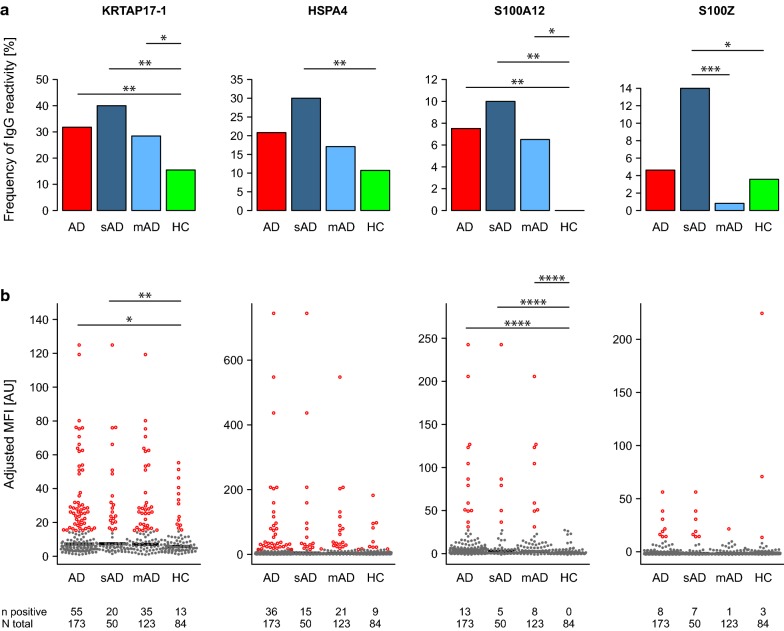
Antigens showing significant difference in IgG-reactivity between healthy controls (HC) and atopic dermatitis patients (AD) or between HC and moderate (mAD) and/or severe (sAD) atopic dermatitis patients when analyzed using antigen bead arrays. **a** The frequency (%) of subjects with IgG-reactivity to KRTAP17-1, HSPA4, S100A12 and S100Z. **b** The adjusted median fluorescence intensity (MFI) of all subjects with positive reactivity highlighted in red. Adjusted MFI was defined as the sample median across all protein fragments subtracted from each signal and divided by the MAD. The number of subjects with positive reactivity out of the total is indicated below each figure. Significant *P* values of pairwise group comparisons indicated by *P < 0.05, **P < 0.01, ***P < 0.001, ****P < 0.0001, Fisher’s exact test (**a**) and Wilcoxon-rank sum test (**b**)

The complete cohort of 173 AD patients (123 mAD and 50 sAD) and 84 HC (Table 1) was analyzed using the targeted antigen suspension bead array covering 148 protein fragments representing 96 proteins (see Fig. 1b and Additional file 1: Table S1). Group comparisons were first made on the level of frequency of subjects with positive IgG-reactivity (Fig. 2a and Table 2). Applying sample- and protein fragment-specific cut-off levels, a significant difference in the frequency of IgG-reactivity was identified for four antigens representing KRTAP17-1; heat shock protein family A (Hsp70) member 4, HSPA4; S100 calcium binding proteins A12, S100A12; and S100Z, differentiating between AD and HC or HC and any of the two subgroups of mAD and sAD (Table 2 and Additional file 1: Table S2). Both KRTAP17-1 and S100Z were included from the untargeted screening where they showed a higher frequency of reactivity in sAD compared to HC. The other two, HSPA4 and S100A12, were not part of the initial screening. The four antigens showed ≥ 10% reactivity in either AD patients or in any of the two subgroups and were selected for further characterization. Positive IgG-reactivity to KRTAP17-1 was observed in significantly higher frequency in AD patients (32%) as well as in mAD (28%) and sAD (40%) compared to HC (15%) (Fig. 2a and Table 2). KRTAP17-1 was represented by a protein fragment covering amino acid 7–38 (Additional file 1: Fig S1A, in red). HPSA4 was found reactive in 21% of AD patients and showed differential reactivity comparing sAD and HC (Fig. 2a and Table 2). Two protein fragments covering different parts of HSPA4 were included in the analysis and reactivity was identified towards the region of amino acid 526–577 (Additional file 1: Fig S1B, in red).

**Table 2:** IgG-reactivity^a^ and adjusted MFI^b^ to four antigens detected in the targeted screening of the complete cohort using antigen bead arrays

Gene	KRTAP17-1	HSPA4	S100A12	S100Z
Gene description	Keratin associated protein 17-1	Heat shock 70 kDa protein 4	S100 calcium binding protein A12	S100 calcium binding protein Z
Ensembl ENSG ID^c^	ENSG00000186860	ENSG00000170606	ENSG00000163221	ENSG00000171643
Uniprot	Q9BYP8	P34932	P80511	Q8WXG8
Protein fragment ID	HPRR3460778	HPRR4220528	HPRR400165	HPRR3460547

Number of IgG-reactive individuals	n (%)	n (%)	n (%)	n (%)
AD patients (N = 173)	55 (32)	36 (21)	13 (8)	8 (5)
Severe^d^ (N = 50)	20 (40)	15 (30)	5 (10)	7 (14)
Moderate^d^ (N = 123)	35 (28)	21 (17)	8 (6)	1 (1)
Healthy controls (N = 84)	13 (15)	9 (11)	0 (0)	3 (4)

*P* values^e^ Calculated on proportion positive individuals				
AD versus HC	0.006	0.054	0.006	1.00
sAD versus mAD	0.15	0.07	0.53	0.0008
sAD versus HC	0.002	0.009	0.006	0.04
mAD versus HC	0.03	0.23	0.02	0.31

Adjusted MFI of individuals	Median (Q1–Q3)	Median (Q1-Q3)	Median (Q1-Q3)	Median (Q1-Q3)
AD patients (N = 173)	7.2 (4.3–18.2)	3.2 (1.3–9.8)	2.6 (0.8–6.5)	− 1.7 (− 2.1 to − 0.6)
Severe^d^ (N = 50)	7.4 (4.6–23.3)	4.0 (1.2–21.3)	2.6 (1.0–6.5)	− 1.8 (− 2.2 to 0.5)
Moderate^d^ (N = 123)	7.1 (4.3–15.9)	3.2 (1.3–8.7)	2.3 (0.8–6.3)	− 1.7 (− 2.1 to − 1.0)
Healthy Controls (N = 84)	5.8 (3.4–10.5)	2.8 (1.1–7.2)	0.8 (0.2–2.0)	− 1.6 (− 2.0 to − 0.2)

*P* values^f^ Calculated on adjusted MFI values				
AD versus HC	0.01	0.36	0.000003	0.16
sAD versus mAD	0.26	0.63	0.50	0.35
sAD versus HC	0.01	0.32	0.00008	0.78
mAD versus HC	0.054	0.48	0.00003	0.09

*AD* atopic dermatitis, *sAD* severe AD, *mAD* moderate AD, *HC* healthy controls, *N* number of individuals, *n* = number of positive individuals

^a^IgG-reactivity was defined using sample-specific cut-offs based on the sample median across protein fragments and the median absolute deviation (MAD), see “Methods” section

^b^Sample median across protein fragments was subtracted from each signal and divided by the MAD, see “Methods” section

^c^Ensembl version 88.38

^d^Objective SCORAD [29], severe AD defined as SCORAD ≥ 41

^e^Fisher’s exact test

^f^Wilcoxon rank-sum test

Interestingly, IgG-reactivity above the cut-off value to S100A12, was only observed in AD patients (8%) with significant differences found between AD or subgroups of AD and HC (Fig. 2a and Table 2). The reactive protein fragment for S100A12 mapped to amino acid 3–92, thereby covering 98% of the full protein sequence (Additional file 1: Fig S1C, in red). Significantly higher reactivity to S100Z was found in sAD patients compared to both HC and mAD (Fig. 2a and Table 2). S100Z was represented by two protein fragments with the reactive region mapped to amino acid 34–71 (Additional file 1: Fig S1D, in red).

The four antigens with differential reactivity were further explored by group comparisons on the level of adjusted MFI (Fig. 2b and Table 2). For KRTAP17-1 and S100A12, most of the significant differences in IgG-reactivity frequency (Fig. 2a) were also observed on the intensity level (Fig. 2b).

**Fig. 3:**
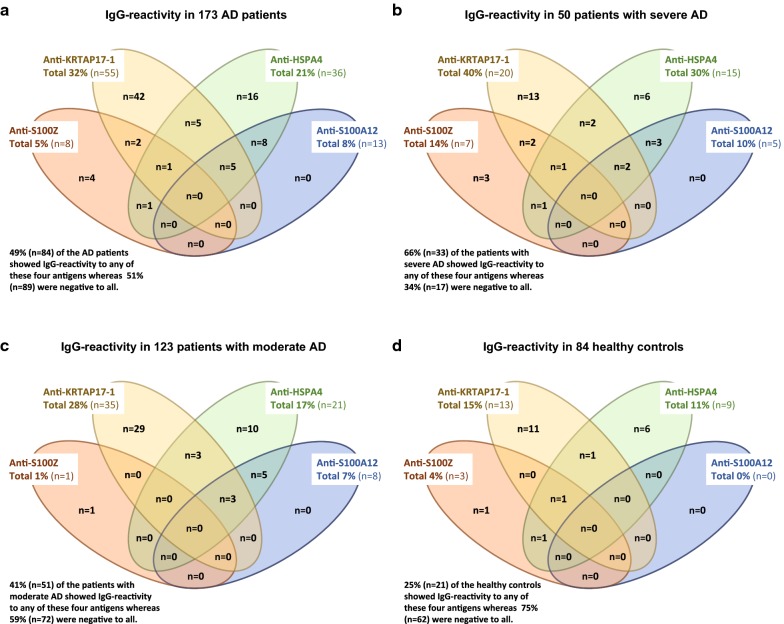
Concordance between IgG-reactivity for the antigens representing KRTAP17-1, HSAP4, S100A12 and S100Z. **a** 173 AD patients, **b** 50 severe AD patients, **c** 123 moderate AD patients, and **d** 84 healthy controls. IgG-reactivity based on analysis of the complete cohort using targeted antigen bead arrays. *AD* atopic dermatitis

**Fig. 4:**
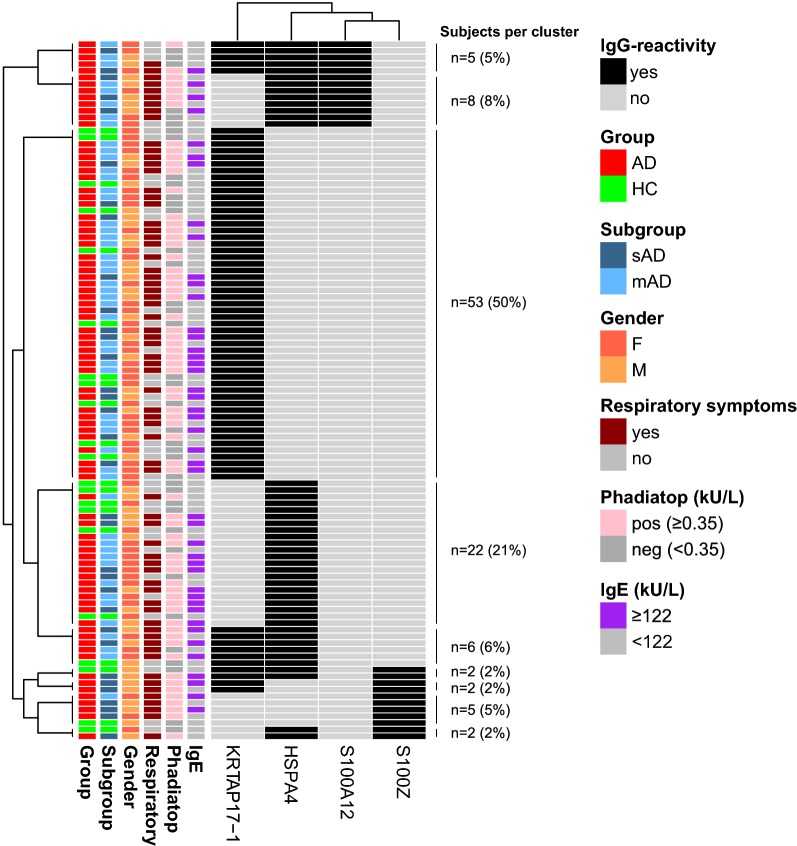
Hierarchical clustering of subjects with IgG-reactivity to the antigens representing KRTAP17-1, HSAP4, S100A12 and S100Z. Clustering of the subjects showing positive IgG-reactivity to any of the four antigens when analyzed using antigen bead arrays. The number of subjects in each cluster and percentage of all subjects with IgG-reactivity are shown. Clinical characteristics annotated in rows. Respiratory symptoms included a history of or current rhinoconjunctivitis and/or asthma. *HC* healthy control, *AD* atopic dermatitis, *mAD* moderate AD, *sAD* severe AD, *F* female, *M* male

Forty-nine percent of the AD patients showed IgG-reactivity to any of the four antigens representing KRTAP17-1, HSPA4, S100A12 and/or S100Z (Fig. 3a). IgG-reactivity to those antigens was more frequent in the sAD patients (66%) than in those with mAD (41%), whereas reactivity only could be found in 25% of HC (Fig. 3b–d). Subjects reactive to any of the four antigens were subjected to hierarchical clustering to show any overlap in reactivity (Fig. 4). Here it was evident that most subjects were reactive to single antigens only, shown by the three clusters corresponding to 76% of all reactive subjects (single reactivity to KRTAP17-1, HSPA4 or S100Z). Notably, this pattern differed for S100A12 where all 13 AD patients reactive to this antigen also showed reactivity to HSPA4 and five of them also to KRTAP17-1 (Fig. 3a). This pattern was the same in sAD and mAD, whereas no HC showed IgG-reactivity to S100A12 (Fig. 3b–d). Four clusters with 17% of the IgG-reactive subjects included reactivity to two antigens (Fig. 4). Reactivity to three antigens was observed in two clusters, corresponding to 7% of the IgG-reactive subjects (Fig. 4). There was no significant difference in the frequency of IgG-reactivity between females and males (Fig. 4) and also no association between age and adjusted MFI for any of the four antigens.

### Associations to allergen specific IgE and total plasma IgE

**Fig. 5:**
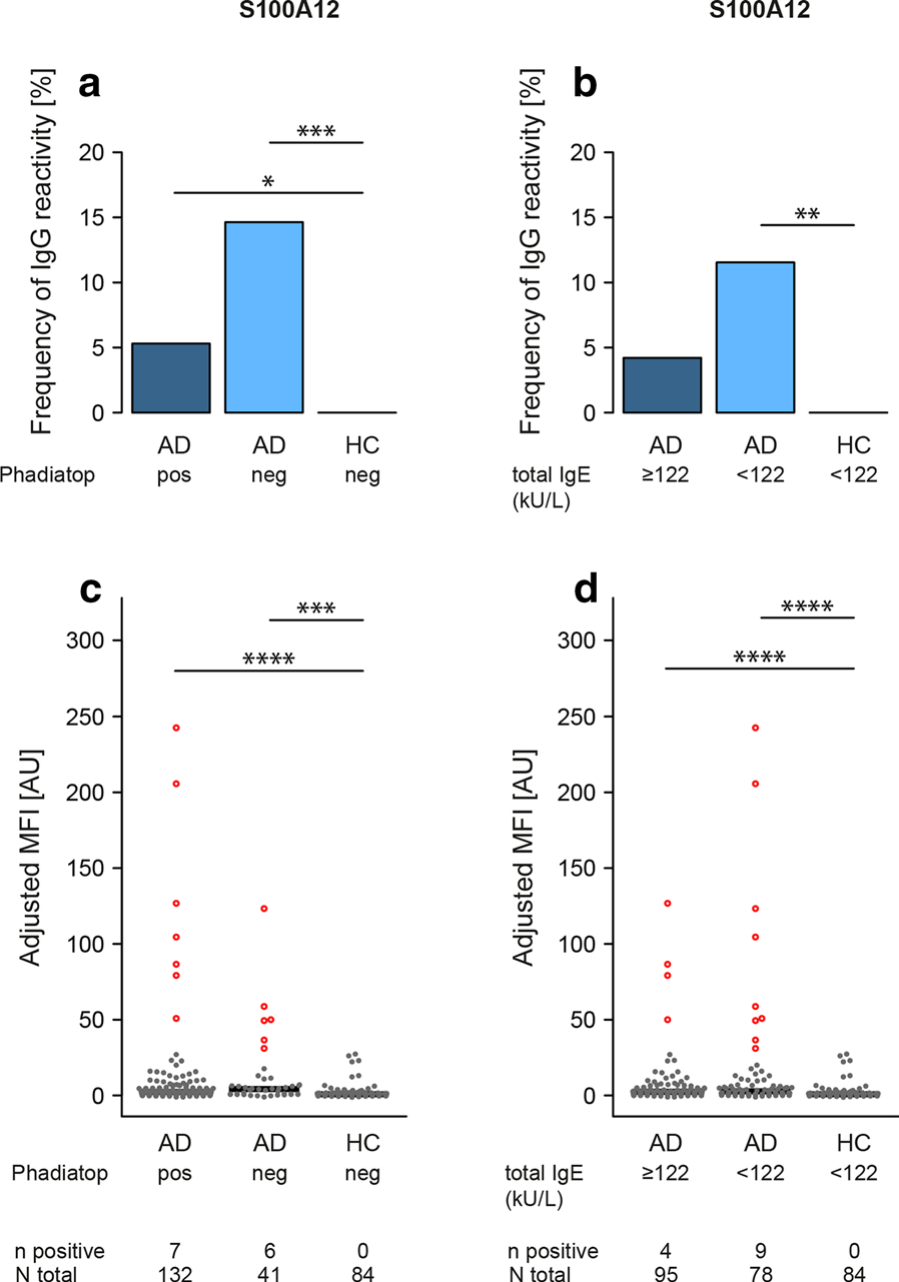
IgG-reactivity to the antigen representing S100A12 subdivided by Phadiatop and total plasma IgE. Antigen showing significant difference in reactivity between healthy controls (HC) and atopic dermatitis cases (AD) or between HC and moderate (mAD) and/or severe (sAD) atopic dermatitis when analyzed using antigen bead arrays. The frequency (%) of subjects with IgG-reactivity to S100A12 subdivided by Phadiatop (**a**) or total plasma IgE (**b**). The adjusted median fluorescence intensity (MFI) of all subjects subdivided by Phadiatop (**c**) or total plasma IgE (**d**). Adjusted MFI was defined by subtracting the sample median across all protein fragments from each signal and dividing the result by the MAD. Subjects with positive reactivity are highlighted in red. The number of subjects with positive reactivity out of the total is indicated below each figure. Significant *P* values of pairwise group comparisons indicated by *P < 0.05, **P < 0.01, ***P < 0.001, ****P < 0.0001, Fisher’s exact test (**a**, **b**) and Wilcoxon-rank sum test (**c**, **d**)

Investigating the relation to allergen specific IgE defined by a positive or negative Phadiatop, revealed significantly higher frequency of IgG-reactivity to KRATP17-1 in AD patients with positive Phadiatop compared to HC as well as higher frequency of IgG-reactivity to S100A12 in AD patients irrespective of Phadiatop compared to HC (Fig. 5a and Additional file 1: Table S3). These differences were also observed when comparing adjusted MFI (Fig. 5b and Additional file 1: Table S3). Subdividing AD patients into subgroups of total plasma IgE lower or higher than 122 kU/L, only showed differences between any of the AD subgroups and HC for KRATP17-1 (Additional file 1: Table S3) and S100A12 (Fig. 5b, d and Additional file 1: Table S3). Details for all four antigens can be found in Additional file 1: Table S3.

### Co-morbidity with respiratory allergic symptoms

**Fig. 6:**
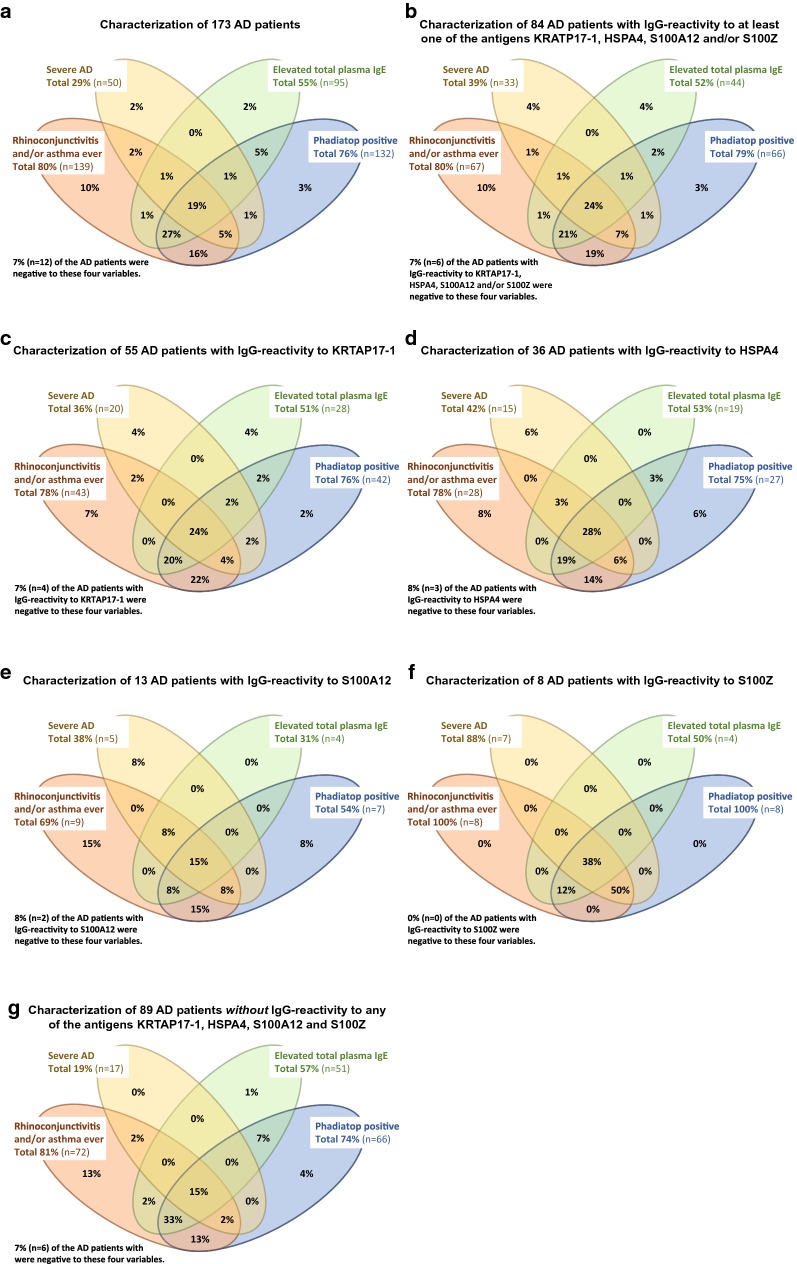
Concordance between rhinoconjunctivitis and/or asthma ever, AD severity (moderate or severe), elevated total plasma IgE and positive Phadiatop. **a** 173 AD patients, **b** 84 AD patients with IgG-reactivity to KRTAP17-1 HSPA4, S100A12 and/or S100Z, **c** 55 AD patients with IgG-reactivity to KRTAP-17, **d** 36 AD patients with IgG-reactivity to HSPA4, **e** 13 AD patients with IgG-reactivity to S100A12, **f** 8 AD patients with IgG-reactivity to S100Z, and **g** 89 AD patients without IgG-reactivity to any of the antigens for KRTAP17-1 HSPA4, S100A12 and/or S100Z. IgG-reactivity based on analysis of the complete cohort using targeted antigen bead arrays, showing frequencies. Elevated total plasma IgE defined as ≥ 122 kU/L. *AD* atopic dermatitis

The majority of patients with AD, irrespective of subgroup, had current or a history of rhinoconjunctivitis and/or asthma (Table 1) in combination with elevated total plasma IgE and a positive Phadiatop (Fig. 6a). This pattern was also observed among the 84 AD patients with IgG-reactivity to at least one of the antigens for KRTAP17-1, HSPA4, S100A12 or S100Z (Fig. 6b). Details for the separate antigens are shown in Fig. 6c–f. The proportion of sAD was significantly larger (*P* < 0.01) among the patients with IgG-reactivity to any of the four antigens compared to the group of patients without any reactivity, whereas the proportion of patients with rhinoconjunctivitis and/or asthma, elevated total plasma IgE and positive Phadiatop was similar (Fig. 6b compared to Fig. 6g).

## Discussion

With the aim to obtain information if autoantibody-based IgG-reactivity can be associated with AD we discovered that auto-reactivity to S100A12 was only observed in AD patients using antigen microarray technology. Three additional proteins KRTAP17-1, HSPA4, and S100Z had a significant increased IgG binding reactivity in plasma samples from AD patients compared to HC and more frequently in the sAD patients than in those with mAD.

Access to large collections of expressed and purified proteins in combination with microarray technology allows for screening of autoantibody reactivity towards hundreds to thousands of antigens in parallel while consuming only small sample volume enabling detection of potential novel autoimmune targets [24]. Planar array formats offer higher theoretical multiplexing in terms of antigens and bead-based formats provide higher sample throughput, making the use of both formats beneficial [40]. The methods used in this study relied on the use of human protein fragments of various lengths and with low similarity to other proteins. Depending on the coverage of the full protein sequence, different conformations are possible and most likely allow for both linear and conformational epitopes to be recognized. Furthermore, as the fragments are expressed as recombinant products in *E. coli*, post-translational modifications (PTMs) such as glycosylation are not present and reactivity towards epitopes dependent on PTMs will not be detected. Utilizing protein fragments instead of full-length proteins mean that for some proteins only a subset of the possible epitopes are present and could result in that potentially autoreactive sequences are not covered or that the fold makes the epitope inaccessible to binding. However, we included several fragments from different parts of the protein when designing the targeted antigen array, thereby increasing the coverage and allowing for closer localization of the reactive region of a protein. This was the case for both KRTAP17-1 and S100Z, where two fragments were included to represent each protein and reactivity identified to only one of them (Fig. S1). Despite differences between the two used array formats as well as increased heterogeneity of individuals when considering the whole cohort, KRTAP17-1 and S100Z were successfully verified in the bead array assay.

S100A12 and S100Z belong to the heterogeneous S100 family of calcium-binding proteins [37]. S100A12, predominantly expressed and secreted by neutrophil granulocytes, is also known as calgranulin C and as extracellular newly identified RAGE-binding protein (EN-RAGE) [41, 42]. S100A12 can trigger pro-inflammatory signaling through TLR4 and exert antimicrobial activity [41]. The reactive protein fragment used for S100A12 in our study mapped to amino acid 3–92, thereby covering 98% of the full protein sequence including the antimicrobial peptide (Fig S1C, in red). Antimicrobial peptides and proteins (AMPs) are effector molecules of the innate defense system and are mainly produced by epithelial cells including keratinocytes [43]. Studies have shown that S100A12, also classified as an epithelial cytokine, is expressed at very low levels in normal epidermis, but it is highly overexpressed in lesions of psoriasis [44, 45], UVB-irradiated skin [46], and tape-stripping of the skin induce fast and long-lasting AMP expression [47]. In addition, S100 proteins are overexpressed in conditions associated with water loss, such as during wound repair why it has been suggested that S100A12 is a potential therapeutic target for dermal scarring [48]. The skin barrier in AD is characterized by dry itchy skin with increased permeability and water loss [1], and with enhanced expression of several AMPs [49, 50] including S100A12 [45]. Regarding serum levels, S100A12 has been found to be the most significant marker for psoriasis disease activity and therapeutic response [45] and as a sensitive and specific diagnostic marker of other inflammatory responses [42], such as asthma where S100A12 provokes mast cell activation and thereby might exacerbate allergic inflammation [51]. Recently, serum levels of S100A12 were found to be differently expressed between four identified AD disease clusters (see Supplemental table E2 in Ref [8]) and were reported significantly upregulated in AD patients versus healthy controls with a correlation between the S100A12 serum levels and SCORAD [52]. In addition, increased serum levels of S100 proteins including S100A12 have been found especially associated with auto-inflammatory diseases, and therefore suggested as promising targets for treatment [53]. In future studies it would thus be interesting to see whether autoantibodies against S100A12 in AD have a mechanistic connection to serum levels of this protein and to increased expression of S100A12 in the skin.

The highest frequency of IgG-reactivity was found against the keratin associated protein 17-1 (KRTAP17-1) among the AD patients (32%) and up to 40% among the sAD patients (Table 2) and with significantly higher frequency compared to HC (15%) for the two subgroups of AD (Fig. 2a). Keratin associated proteins (KRTAPs) are one of the main structural components of hair, and are expressed in distinct cell populations of the hair follicle involved in hair formation, where KRTAP17-1 shows strong expression in the upper hair cuticle [54]. The hair cuticle structure is severely damaged and distorted in lesional and non-lesional AD [55]. This could result in an increased exposure of altered structural hair components to the immune system inducing autoantibody production.

The majority of the AD patients had also current or a history of rhinoconjunctivitis and/or asthma in combination with elevated total plasma IgE and were positive in Phadiatop with a higher proportion in the sAD compared with the mAD group (Table 1). However, cluster analysis did not reveal any particular subgroup associated with increased IgG-reactivity to any of the antigens for KRTAP17-1, HSPA4, S100A12 and/or S100Z besides more frequent IgG-reactivity in the sAD than in the mAD group. The data would thus, rather suggest that IgG-reactivity to any of these four antigens reflects AD severity rather than co-morbidity with respiratory allergic symptoms.

IgG-reactivity to KRTAP17-1, HSPA4 and S100Z, but not to S100A12 was also observed in 21 of the 84 HC (25%) (Fig. 3d) compared to 49% of the 173 AD patients reactive to any of the four antigens (Fig. 3a). Autoantibodies are not necessarily pathogenic, but often observed in healthy individuals [56]. It has been suggested that “natural” IgG autoantibodies might participate in an adaptive debris-clearance mechanism [57], which could be the case for the three autoantibodies we found both in HC and in AD patients. Pathologic autoantibodies can induce disease through several different pathways [56]. Whether the autoantibodies against S100A12 we detected in a subgroup of AD patients are protective, pathogenic or merely an epiphenomenon remains to be determined. One hypothesis could be that production of these autoantibodies is induced by the chronic inflammation with enhanced expression of S100A12 in the skin and that they could either exhibit anti-inflammatory or pro-inflammatory activities connected to the function of S100A12. A mechanism could be that the autoantibodies possess a pathogenic function by binding to and clearing S100A12, thereby preventing the protein from exerting its antimicrobial function and potentially contributing to increased susceptibility to skin infections. Further studies in additional sample cohorts are needed in addition to the characterization of isotypes and subclasses of autoantibodies in AD and the elucidation of their functional activity in vivo.

## Conclusions

We have utilized an untargeted array-based screening on more than 1100 protein fragments followed by the analysis of 257 human AD and HC plasma samples in an antigen suspension bead array representing 96 selected proteins. Introducing array methodologies for detailed phenotype characterization of the autoantibody IgG profile in patients with AD have here provided the possibility for identification of novel candidate autoantigens. We have found a subgroup of AD patients with IgG-reactivity to four autoantigens, among those S100A12 with antimicrobial activity. Further characterization of their implication for AD could be part of the need to develop personalized strategies for prevention and management to break, terminate or reverse the natural course of this chronic disease.

## Additional file

**Additional file 1.** Supplementary Methods, Table S1, Table S2, Table S3 and Fig S1.

## Abbreviations

AD: atopic dermatitis
AMPs: antimicrobial peptides and proteins
HC: healthy controls
His6-ABP: N-terminal hexa-histidine albumin binding protein
HSPA4: heat shock protein family A (Hsp70) member 4
KRTAP17-1: keratin associated protein 17-1
mAD: moderate atopic dermatitis
MAD: median absolute deviation
MFI: median fluorescence intensity
PTM: post-translational modifications
S100A12: S100 calcium binding protein A12
S100Z: S100 calcium binding protein Z
sAD: severe atopic dermatitis
SCORAD: scoring atopic dermatitis
