# Supplementary material for: The antimicrobial protein S100A12 identified as a potential autoantigen in a subgroup of atopic dermatitis patients
Source: Clin Transl Allergy. 2019 Jan 31;9:6. doi: 10.1186/s13601-019-0240-4 (PMC6354350; doi:10.1186/s13601-019-0240-4)
Supplement: Supplementary file 1 — Additional file 1. Supplementary Methods, Table S1, Table S2, Table S3 and Fig S1. [file 13601_2019_240_MOESM1_ESM.docx]

# Supplementary material to:

**The antimicrobial protein S100A12 identified as a potential autoantigen in a subgroup of atopic dermatitis patients**

Maria Mikus, PhD^1^, Catharina Johansson, PhD^2^, Nathalie Acevedo, MD, PhD^2,3^, Peter Nilsson, PhD^1^, and Annika Scheynius, MD, PhD^2,4*^

^1^ Affinity Proteomics, SciLifeLab, Department of Protein Science, KTH Royal Institute of Technology, Stockholm, Sweden

^2^ Department of Clinical Science and Education, Karolinska Institutet, and Sachs' Children and Youth Hospital, Södersjukhuset, Stockholm, Sweden

^3^ Institute for Immunological Research, University of Cartagena, Cartagena, Colombia

^4^ Clinical Genomics, SciLifeLab, Stockholm, Sweden

* Corresponding author:

Annika Scheynius, MD, PhD,

Department of Clinical Science and Education,

Södersjukhuset,

Karolinska Institutet,

SE-11883 Stockholm, Sweden,

+46 (0)706057927,

E-mail: [annika.scheynius@ki.se](mailto:annika.scheynius@ki.se)

# SUPPLEMENTARY METHODS

## Planar antigen microarrays

## In the screening phase, 1.5 µl of plasma from 80 subjects (40 sAD patients and 40 HC patients, Fig 1A) were diluted 1:250 in assay buffer using a liquid handler (SELMA, CyBio, Jena, Germany). The sAD patients were individually age- and gender-matched with the HC (12 females and 28 males in each group, age median (range) were 31 (18-65) years in sAD and 31 (18-64) years in HC). The assay buffer contained 3% (w/v) bovine serum albumin (BSA, Saveen Werner AB, Limhamn, Sweden), 5% (w/v) nonfat milk powder (Semper, Sundbyberg, Sweden) and 0.1% Tween-20 in PBS (Medicago AB, Uppsala, Sweden) (PBS-T), supplemented with 160 µg/ml His6-ABP. Samples were pre-incubated for 1 h at room temperature on a shake table before applied to the subarrays (60 µl) in a randomized fashion (equal numbers of AD patients and age- and gender matched HC per subarray) using a liquid handler (EVO150, Tecan Group, Männedorf, Switzerland) and incubated for 2 h. Slides were washed in 0.1% PBS-T and incubated with hen anti-His6-ABP IgY (1:25000, Agrisera, Vännäs, Sweden) for 45 min. After washing, a mixture of Alexa Fluor 647-conjugated goat F(ab')2 anti-human IgG Fcγ specific (1:25000, Jackson ImmunoResearch, West Grove, PA, USA) and Alexa Fluor 555-conjugated goat anti-hen IgG (1:60000, Invitrogen, Thermo Fisher Scientific) was applied for 60 min. After washing, slides were dried and scanned (Agilent G2565BA array scanner, Agilent Technologies Inc., Santa Clara, CA, USA) followed by image analysis (GenePix Pro 5.1, Molecular Devices, Sunnyvale, CA, USA) , reporting median fluorescence intensity (MFI) for each array spot.

## Antigen suspension bead arrays

In the targeted analysis phase, 5x10^5^ beads were washed in phosphate buffer (0.1 M NaH_2_PO_4_) before activated with 0.5 mg 1‑ethyl‑3‑(3‑dimethylaminopropyl)carbodiimide hydrochloride (EDC, Pierce, Thermo Fisher Scientific, Waltham, MA, USA) and 0.5 mg N‑hydroxysulfosuccinimide (S-NHS, Pierce) in phosphate buffer for 20 min. Following washing of beads in 2‑(N‑Morpholino)ethanesulfonic acid (MES, 0.05 M) using a plate washer (EL406, Biotek, Winooski, VT, USA), protein fragments (40 µg/ml in MES) were added to beads and incubated for 2 h. After a final wash in 0.05% PBS-T, beads were stored overnight in a blocking reagent for ELISA (Roche Diagnostics, Mannheim, Germany) before combined into a multiplex suspension bead array. Successful immobilization of protein fragments was confirmed using hen anti-His6-ABP IgY (Agrisera) and R-phycoerythrin conjugated donkey F(ab')2 anti-hen IgY (Jackson ImmunoResearch). Before the assay, 50 µl of plasma samples were randomly distributed in 96-well microtiter plates (Thermo Fisher Scientific), controlling for an even distribution of AD patients and HC across plates with regard to numbers, gender and age. In addition, a buffer blank in duplicates and a pool of all samples were aliquoted in triplicates across each plate for determination of technical variation. For the assay, 1 µl of plasma was diluted 1:250 as described above for the planar antigen array using a liquid handler (SELMA, CyBio) and 45 µl diluted samples were incubated with the beads in a 384-well plate (Greiner Bio-One, Kremsmünster, Austria) for 2 h. After washing with 0.05% PBS-T using a plate washer, bound IgG were crosslinked using 0.2% paraformaldehyde and later detected by R-phycoerythrin conjugated goat F(ab')2 anti-human IgG Fcγ specific (1:750, 30 min, Jackson ImmunoResearch). Read-out was performed in a FlexMAP3D system (Luminex corp.), reporting MFI for each bead identity.

# SUPPLEMENTARY TABLES AND FIGURES

**Table S1.** List of the 148 protein fragments included in the targeted analysis using antigen bead arrays. This selection of protein fragments was based on findings from the untargeted screening process on planar antigen arrays. In addition, protein fragments representing targets from literature [1-3] and mRNA [4] and DNA methylation [4] screenings were also included. The four antigens identified to be reactive in the targeted analysis are highlighted in red.

| **Gene** | **Gene description** | **Uniprot** | **ENSG** | **Protein fragment ID** | **Sequence** | **Fragment length (aa)** | **Source** |
| --- | --- | --- | --- | --- | --- | --- | --- |
| ARRB1 | arrestin beta 1 | P49407 | ENSG00000137486 | HPRR3460202 | LKGMKDDKEEEEDGTGSPQLNNR | 23 | Screening  (≥10% reactivity difference) |
| ARRB1 | arrestin beta 1 | P49407 | ENSG00000137486 | HPRR3460203 | PKPKEEPPHREVPENETPVDTNL | 23 | Screening  (different region) |
| C2orf61 | chromosome 2 open reading frame 61 | Q8N801 | ENSG00000239605 | HPRR3690182 | HEGPGPGHYNVKMPPTSSVTSCFQSRVPRFLPSCSKTPGPGAYTTLRQFPKQSPTIAKMGQEHSLFFNNNNWL | 73 | Screening  (≥10% reactivity difference) |
| C2orf61 | chromosome 2 open reading frame 61 | Q8N801 | ENSG00000239605; ENSG00000273269 | HPRR3400242 | VGGESFITASKPAQKTSSFEREGWWRIALTDTPIPGTYHLKTFIEESLLNPVIATYNFKNEGRKKPPLVQRNNPVLNDLPQYMPPDFLDLL | 91 | Screening  (different region) |
| CPNE1 | copine 1 | Q99829 | ENSG00000214078 | HPRR3560103 | KNNLNPTWKRFSVPVQHFCGGNPSTPIQVQCSDYDSDGS | 39 | Screening  (≥10% reactivity difference) |
| CPNE1 | copine 1 | Q99829 | ENSG00000214078 | HPRR3560102 | LAQTVLAEVPTQLVSYFRAQGWAPLKPLPPSAKDPAQA | 38 | Screening  (different region) |
| CPNE1 | copine 1 | Q99829 | ENSG00000214078 | HPRR3560104 | DLIGTFHTSLAQLQAVPAEFECIHPEKQQK | 30 | Screening  (different region) |
| DGKG | diacylglycerol kinase gamma | P49619 | ENSG00000058866 | HPRR2960061 | TEGASNSEANSADTNIQNADNATKADEACAPDTESNMAEKQAPAEDQVAATPLEPPVPRSSSSESPVVYLKDV | 73 | Screening  (≥10% reactivity difference) |
| DGKG | diacylglycerol kinase gamma | P49619 | ENSG00000058866 | HPRR2960060 | HCVWCRMTFHRKCELSTLCDGGELRDHILLPTSICPITRDRPGEKSDGCVSAKGELVMQYKII | 63 | Screening  (different region) |
| DGKG | diacylglycerol kinase gamma | P49619 | ENSG00000058866 | HPRR2960062 | MGEERWVSLTPEEFDQLQKYSEYSSKKIKDALTEFNEGGSLKQYDPHEPISYDVFKLFMRAYLEVDLPQPLSTHLFLA | 78 | Screening  (different region) |
| FAM9C | family with sequence similarity 9 member C | Q8IZT9 | ENSG00000187268 | HPRR3690171 | ETDEHTGVDTKELEDIAADIKEHLAAKRKRIEKIAKACSEIKNRIK | 46 | Screening  (*P* <0.05) |
| FAM9C | family with sequence similarity 9 member C | Q8IZT9 | ENSG00000187268 | HPRR4050353 | MAAKDQLEVQVMAAQEMELAGKDPVSHEHEERKPVTETKEG | 41 | Screening  (different region) |
| GPR180 | G protein-coupled receptor 180 | Q86V85 | ENSG00000152749 | HPRR3140926 | KLSKAQLTMTMNQTEHNLTVSQIPSPQTWHVFYADKYTCQDDKENSQVEDIPFEMVLLNPDAEGNPFDHFSAGES | 75 | Screening  (≥10% reactivity difference) |
| GPR180 | G protein-coupled receptor 180 | Q86V85 | ENSG00000152749 | HPRR3140925 | QRIGHFEFHGDHALLCVRINNIAVAVGKEAKLYLFQAQEWLKLQQSSHGYSCSE | 54 | Screening  (different region) |
| GPR180 | G protein-coupled receptor 180 | Q86V85 | ENSG00000152749 | HPRR3140927 | RLFLSHSLYWEVSSLSSVTLPLTISSGHK | 29 | Screening (different region) |
| HHIPL1 | HHIP like 1 | Q96JK4 | ENSG00000182218 | HPRR3450164 | DVDRKERGLPYGIPPDNPFVGDPAAQPEVYALGVRNMWRCSFDRGDPSSGT | 51 | Screening  (≥10% reactivity difference) |
| HHIPL1 | HHIP like 1 | Q96JK4 | ENSG00000182218 | HPRR3450163 | GRLMSLQENPGTGQWQYSEICMGHGQTCEFPGLINNYYPYIISFG | 45 | Screening (different region) |
| KRTAP17-1 | keratin associated protein 17-1 | Q9BYP8 | ENSG00000186860 | HPRR3460778 | DCFTCCTQEQNCCEECCCQPGCCGCCGSCCGC | 32 | Screening  (≥10% reactivity difference) |
| MFSD2B | major facilitator superfamily domain containing 2B | A6NFX1 | ENSG00000205639 | HPRR3400359 | MLPDVVDDFQLQHRHGPGLETIFYSSY | 27 | Screening  (≥10% reactivity difference) |
| MFSD2B | major facilitator superfamily domain containing 2B | A6NFX1 | ENSG00000205639 | HPRR3400360 | VGSTPKTPSRDASSRLSLRRRTSYSL | 26 | Screening (different region) |
| MICU1 | mitochondrial calcium uptake 1 | Q9BPX6 | ENSG00000107745 | HPRR3020124 | IFYTLGECGLISFSDYIFLTTVLSTPQRNFEIAFKMFDLNGDGEVDMEEFEQVQSIIRSQTSMGMRHRDRPTTGNTLKSGLCSALTTY | 88 | Screening  (≥10% reactivity difference) |
| MICU1 | mitochondrial calcium uptake 1 | Q9BPX6 | ENSG00000107745 | HPRR3020125 | LKGKLTIKNFLEFQRKLQHDVLKLEFERHDPVDGRITERQFGGMLLAYSGVQSKKLTAMQRQLKKHFKEGKGLTFQEVENFFTFL | 85 | Screening  (different region) |
| MRPL43 | mitochondrial ribosomal protein L43 | Q8N983 | ENSG00000055950 | HPRR3590069 | CCVPRVVAEYLNGAVREESIHCKSVEEISTLVQKLADQSGLDVIRIRKPFHTDNPSIQGQWHPFTNKPTTF | 71 | Screening  (≥10% reactivity difference) |
| MRPL43 | mitochondrial ribosomal protein L43 | Q8N983 | ENSG00000055950 | HPRR3590068 | GTPSRFLASVLHNGLGRYVQQLQRLSFSVSRDGASSRGAREFVEREVIDFARRNPGVVIYVNSRPC | 66 | Screening (different region) |
| NXT1 | nuclear transport factor 2 like export factor 1 | Q9UKK6 | ENSG00000132661 | HPRR3690076 | SGQESLSEFFEMLPSSEFQISVVD | 24 | Screening  (≥10% reactivity difference) |
| OAF | out at first homolog | Q86UD1 | ENSG00000184232 | HPRR3050954 | RFWLEQGVDSSVFEALPKASEQAELPRCRQVGDRGKPCVCHYGLSLAWYPCMLKYCHSRDRPTPYKCGIRSCQKSYSFDFYVPQ | 84 | Screening  (≥10% reactivity difference) |
| OAF | out at first homolog | Q86UD1 | ENSG00000184232 | HPRR4050009 | SFTADFKKDVKVFRALILGELEKGQSQFQALCFVTQLQHNEIIPSEAMAKLRQKNPRAVRQAEEVRGLEHLHMDV | 75 | Screening (different region) |
| OR2T11 | olfactory receptor, family 2, subfamily T, member 11 (gene/pseudogene) | Q8NH01 | ENSG00000183130 | HPRR3580070 | MTNTSSSDFTLLGLLVNSEAA | 21 | Screening  (*P* <0.05) |
| OR2T11 | olfactory receptor, family 2, subfamily T, member 11 (gene/pseudogene) | Q8NH01 | ENSG00000183130 | HPRR4080070 | RNKDVIGAFKKVFACCSSARKVATSDA | 27 | Screening (different region) |
| RAB26 | RAB26, member RAS oncogene family | Q9ULW5 | ENSG00000167964 | HPRR3420131 | SGTALSGPDAPPNGPLQPGRPSLGGGVDFYDVAFKVMLV | 39 | Screening  (≥10% reactivity difference) |
| RAB26 | RAB26, member RAS oncogene family | Q9ULW5 | ENSG00000167964 | HPRR3420132 | AKELKQRSMKAPSEPRFRLHDYVKREGR | 28 | Screening (different region) |
| RUFY2 | RUN and FYVE domain containing 2 | Q8WXA3 | ENSG00000204130 | HPRR3020918 | EELAIAKNNIIKLQEENHQLRSENKLILMKTQQHLEVTKVDVETELQTYKHSRQGLDEMYNEARRQLRDESQLRQDVENELAVQVSMKHEIEL | 93 | Screening  (≥10% reactivity difference) |
| RUFY2 | RUN and FYVE domain containing 2 | Q8WXA3 | ENSG00000204130 | HPRR3020917 | EKAQMEAEDEDEKYLQECLSKSDSLQKQISQKEKQLVQLETDLKIEKEWRQTLQE | 55 | Screening (different region) |
| S100A3 | S100 calcium binding protein A3 | P33764 | ENSG00000188015 | HPRR3420667 | MARPLEQAVAAIVCTFQEYAGRCGDKYKLCQAELKELLQK | 40 | Screening  (*P* <0.05) |
| S100A3 | S100 calcium binding protein A3 | P33764 | ENSG00000188015 | HPRR3420666 | KELATWTPTEFRECDYNKFMSVLDTNKDCEVDFVEYVRSLACLCLYCHEYFKD | 53 | Screening (different region) |
| S100Z | S100 calcium binding protein Z | Q8WXG8 | ENSG00000171643 | HPRR3460547 | LKLLLQRELTEFLSCQKETQLVDKIVQDLDANKDNEVD | 38 | Screening  (≥10% reactivity difference) |
| S100Z | S100 calcium binding protein Z | Q8WXG8 | ENSG00000171643 | HPRR3460548 | MPTQLEMAMDTMIRIFHRYSGKARKRFKLS | 30 | Screening (different region) |
| SNX11 | sorting nexin 11 | Q9Y5W9 | ENSG00000002919 | HPRR3500048 | SEVPSLESPTLPPLSSPLCCDFGRPKEGTSTLQSVRRAVGGDHAVPLDHGQLETV | 55 | Screening  (≥10% reactivity difference) |
| SNX11 | sorting nexin 11 | Q9Y5W9 | ENSG00000002919 | HPRR3500049 | LFLQSQLSVPEIEACVQGRSTMTVSDAILRYAMSNCGWAQEERQSSSHLAKGDQPKSCCFLPRSGRRSSP | 70 | Screening (different region) |
| TEN1 | TEN1, CST complex subunit; TEN1-CDK3 readthrough (NMD candidate) | Q86WV5 | ENSG00000257949; ENSG00000261408 | HPRR3470283 | KLVEPFHAQVGSLYIVLGELQHQQDRGSVVKARVLTCVEGMNLPLLEQAIREQRLYKQER | 60 | Screening  (≥10% reactivity difference) |
| TEN1 | TEN1, CST complex subunit; TEN1-CDK3 readthrough (NMD candidate) | Q86WV5 | ENSG00000257949; ENSG00000261408 | HPRR3470282 | MMLPKPGTYYLPWEVSAGQVPDGSTLRTFGRLCLYDMIQSRVTLMAQHGSDQHQVLVCTK | 60 | Screening (different region) |
| TRIM52 | tripartite motif containing 52 | Q96A61 | ENSG00000183718 | HPRR3790297 | EDEEAVGAMDGWDGSIREVLYRGNADEELFQDQDDDELWLGDSGITNWDNVDYMWDEE | 58 | Screening  (≥10% reactivity difference) |
| TRIM52 | tripartite motif containing 52 | Q96A61 | ENSG00000183718 | HPRR3790298 | EEVVQEYQEIKLETTLVGILQIEQESIHSKAYNQ | 34 | Screening (different region) |
| UTP14A;UTP14C | UTP14A small subunit processome component; UTP14C, small subunit processome component | Q9BVJ6; Q5TAP6 | ENSG00000156697; ENSG00000253797 | HPRR3360164 | ARMMERMSLKHQNSGKWAKSKAIMAKYDLEARQAMQEQLAKNKELTQKLQVASESEEE | 58 | Screening  (≥10% reactivity difference) |
| UTP14A;UTP14C | UTP14A small subunit processome component; UTP14C, small subunit processome component | Q9BVJ6; Q5TAP6 | ENSG00000156697; ENSG00000253797 | HPRR3360161 | RVQTLEELEELGKEDCFQNKELPRPVLEGQQSERTPNNRPDAPKEKKEKEQ | 51 | Screening (different region) |
| ZBTB25 | zinc finger and BTB domain containing 25 | P24278 | ENSG00000089775 | HPRR3700073 | IGLDDGTADQQRACPATQALEEHQKPPVSIKQERCDPESVISQSHPSPSSEVTGPTFTENSVKIHLCHYCGERFDSRSNLR | 81 | Screening  (≥10% reactivity difference) |
| ZBTB25 | zinc finger and BTB domain containing 25 | P24278 | ENSG00000089775 | HPRR3700071 | ICGHKFPRKSQLLEHMYTHKGKSYRYNRCQRFGNALAQRFQPYCDSWSDVSLKSSRLSQEHLDLPCALESELTQENVDTILVE | 83 | Screening (different region) |
| CAMP | cathelicidin antimicrobial peptide |  | ENSG00000164047 | HPRR1951441 | RSSDANLYRLLDLDPRPTMDGDPDTPKPVSFTVKETVCPRTTQQSPEDCDFKKDGLVKRCMGTVTLNQARGSFDISCDKDNKRFALLGDFFRKSKEKIGKEFKRIVQRIKDFLRNLVPR | 119 | mRNA screening[4] |
| CD58 | CD58 molecule | P19256 | ENSG00000116815 | HPRR1950575 | SQQIYGVVYGNVTFHVPSNVPLKEVLWKKQKDKVAELENSEFRAFSSFKNRVYLDTVSGSLTIYNLTSSDEDEYEMESPNITDTMKFFLYVLESLPSPTLTCALTNGSIEVQCMIPEHYNSHRGLIMYSWDCPMEQCKRNSTSIYFKMENDLPQKIQCTLSNPLFNTTSSIILTTCIPSSGHSRHRY | 187 | mRNA screening[4] |
| CD58 | CD58 molecule | P19256 | ENSG00000116815 | HPRR2990063 | IMYSWDCPMEQCKRNSTSIYFKMENDLPQKIQCTLSNPLFNTTSSIILTTCIPSSGHSRHRYA | 63 | mRNA screening[4] |
| CD58 | CD58 molecule | P19256 | ENSG00000116815 | HPRR2990064 | DTVSGSLTIYNLTSSDEDEYEMESPNITDTMKFFLYVLESLPSPTLTCALTNGSIEVQCMIPEHYNSHRGL | 71 | mRNA screening[4] |
| CD58 | CD58 molecule | P19256 | ENSG00000116815 | HPRR4420102 | CFSQQIYGVVYGNVTFHVPSNVPLKEVLWKKQKDKVAELENSEFRAFSSFKNRVYLD | 57 | mRNA screening[4] |
| CFL1 | cofilin 1 | P23528 | ENSG00000172757 | HPRR3290106 | HELQANCYEEVKDRCTLAEKLGGSAVISL | 29 | mRNA screening[4] |
| CFL1;CFL2;DSTN | cofilin 1; cofilin 2; destrin actin depolymerizing factor | P23528; Q9Y281; P60981 | ENSG00000172757; ENSG00000165410; ENSG00000125868 | HPRR4290591 | FWAPESAPLKSKMIYASSKDAIKKKL | 26 | mRNA screening[4] |
| CRNN | cornulin | Q9UBG3 | ENSG00000143536 | HPRR1420099 | YDRQAESQSQERISPQIQLSGQTEQTQKAGEGKRNQTTEMRPERQPQTREQDRAHQTGETVTGSGTQTQAGATQTVEQDSSHQTGRTSKQTQEATNDQNR | 100 | Literature[2] |
| DEFA4 | defensin alpha 4 | P12838 | ENSG00000164821 | HPRR3760857 | SSALQVSGSTRGMVCSCRLVFCRRTELRVGNCLIGGVSFTYCCTRVD | 47 | mRNA screening[4] |
| DEFA4 | defensin alpha 4 | P12838 | ENSG00000164821 | HPRR3970309 | ARGDEAPGQEQRGPEDQDISISFAWDK | 27 | mRNA screening[4] |
| EXT1 | exostosin glycosyltransferase 1 | Q16394 | ENSG00000182197 | HPRR1370208 | DSSVHISPRQKRDANSSIYKGKKCRMESCFDFTLCKKNGFKVYVYPQQKGEKIAESYQNILAAIEGSRFYTSDPSQACLFVLSLDTLDRDQLSPQYVHNLRS | 102 | DNA methylation screening[4] |
| EXT1 | exostosin glycosyltransferase 1 | Q16394 | ENSG00000182197 | HPRR1370209 | EDVGFDIGQAMLAKASISTENFRPNFDVSIPLFSKDHPRTGGERGFLKFNTIPPLRKYMLVFKGKRYLTGIGSDTRNALYHVHNGEDVVLLTTCKHGKDWQKHKDSRCDRDNTEYEKYDYREMLHNATFCL | 131 | DNA methylation screening[4] |
| FKBP4 | FK506 binding protein 4 | Q02790 | ENSG00000004478 | HPRR4160063 | DFELARADFQKVLQLYPNNKAAKTQLAVCQQRIRRQLAREKKLYANMFERLAEEENKAKAEASSGDHPTDTEMKEEQKSNTAGSQSQVE | 89 | mRNA screening[4] |
| FKBP4 | FK506 binding protein 4 | Q02790 | ENSG00000004478 | HPRR520156 | IATMKVGEVCHITCKPEYAYGSAGSPPKIPPNATLVFEVELFEFKGEDLTEEEDGGIIRRIQTRGEGYAKPNEGAIVEVALEGYYKDKLFDQRELRFEIGEGENLDLPYGLERAIQRMEKGE | 122 | mRNA screening[4] |
| FLG | filaggrin | P20930 | ENSG00000143631 | HPRR2551092 | SERWSGSASRNHLGSAWEQSRDGSRHPGSHHEDRAGHGHSADSSRQSGTRHTESSSRGQAASSHEQARSSAGERHGSRHQLQSA | 84 | Literature[2] |
| FLG | filaggrin | P20930 | ENSG00000143631 | HPRR2551093 | SADSSRQSGTPHAETSSGGQAASSHEQARSSPGERHGSRHQQSADSSRHSGIPRRQASSAVRDSGHWGSSGSQASDSEGHSEESDTQSVSGHGQDGPHQQSHQESAR | 107 | Literature[2] |
| FLG | filaggrin | P20930 | ENSG00000143631 | HPRR2551094 | RKRLSERLEEKEDNEEGVYDYENTGRMTQKWIQSGHIATYYTIQDEAYDTTDSLLEENKIYERSRSSDGKSSSQVNRSRHENTSQVPLQESR | 92 | Literature[2] |
| FLG2 | filaggrin family member 2 | Q5D862 | ENSG00000143520 | HPRR2551079 | CGYSNSSGCGRPQNASSSCQSHRFGGQGNQFSYIQSGCQSGIKGGQGHGCVSGGQPSGCGQPESNPCSQSYSQRGYGARENGQPQNCGGQWRTGSSQSSCCG | 102 | Literature[2] |
| HLA-A;HLA-B;HLA-C;HLA-E;HLA-F;HLA-G | major histocompatibility complex, class I, A; B; C; E; F; G | P04439+P13746+P16188; P01889+P30480+P30486+Q31610+Q31612+Q29836; P10321; NA; P30511; P17693 | ENSG00000206503; ENSG00000234745; ENSG00000204525; ENSG00000204592; ENSG00000204642; ENSG00000204632 | HPRR3430084 | QTQDTELVETRPAGDGTFQKWAAVVVPSG | 29 | mRNA screening[4] |
| HLA-A;HLA-B;HLA-C;HLA-E;HLA-F;HLA-G | major histocompatibility complex, class I, A; B; C; E; F; G | P04439+P13746+P16188; P01889+P30480+P30486+Q31610+Q31612+Q29836; P10321; NA; P30511; P17693 | ENSG00000206503; ENSG00000234745; ENSG00000204525; ENSG00000204592; ENSG00000204642; ENSG00000204632 | HPRR3430085 | DHEATLRCWALGFYPAEITLTWQRD | 25 | mRNA screening[4] |
| HLA-A;HLA-B;HLA-C;HLA-E;HLA-F;HLA-G | major histocompatibility complex, class I, A; B; C; E; F; G | P04439+P13746+P16188; P01889+P30480+P30486+Q31610+Q31612+Q29836; P10321; NA; P30511; P17693 | ENSG00000206503; ENSG00000234745; ENSG00000204525; ENSG00000204592; ENSG00000204642; ENSG00000204632 | HPRR4470247 | YFSAAVSRPGRGEPRFIAMGYVDDTQFVRFDSDSA | 35 | mRNA screening[4] |
| HMGB1;HMGB2 | high mobility group box 1; high mobility group box 2 | P09429; P26583 | ENSG00000189403; ENSG00000164104 | HPRR430077 | KSKFEDMAKSDKARYDREMKNYVPPKGDKKGKKKDPNAPKRPPSAFFLFCSEHRPKIKSEHPGLSIGDTAKKLGEMWSEQSAKDKQPYEQKAAKLKEKYE | 100 | mRNA screening[4], Literature[1] |
| HMGB1;HMGB2;HMGB3 | high mobility group box 1; high mobility group box 2; high mobility group box 3 | P09429; P26583; O15347 | ENSG00000189403; ENSG00000164104; ENSG00000029993 | HPRR3430022 | KGDPKKPRGKMSSYAFFVQTCREEHKKK | 28 | mRNA screening[4], Literature[1] |
| HMGB2 | high mobility group box 2 | P26583 | ENSG00000164104 | HPRR3440063 | AKGKSEAGKKGPGRPTGSKKKNEP | 24 | mRNA screening[4] |
| HMGN2;HMGN4 | high mobility group nucleosomal binding domain 2; high mobility group nucleosomal binding domain 4 | P05204; O00479 | ENSG00000198830; ENSG00000182952 | HPRR3360098 | PKRKAKGDAKGDKAKVKDEPQ | 21 | mRNA screening[4] |
| HMGN2;HMGN4 | high mobility group nucleosomal binding domain 2; high mobility group nucleosomal binding domain 4 | P05204; O00479 | ENSG00000198830; ENSG00000182952 | HPRR4050064 | KKGEKLPKGRKGKADAGKDGNNPAKNRDA | 29 | mRNA screening[4] |
| HRNR | hornerin | Q86YZ3 | ENSG00000197915 | HPRR2552125 | WSAGENDSYSRNVRGSLKPGTESISRRLSFQRDFSGQHNSYSGQSSSYGEQNSDSHQSSGRGQCGSGS | 68 | Literature[2] |
| HRNR | hornerin | Q86YZ3 | ENSG00000197915 | HPRR2552126 | RDHNKKVDFTEYLLMIFKLVQARNKIIGKDYCQVSGSKLRDDTHQHQEEQEETEKEENKRQES | 63 | Literature[2] |
| HRNR | hornerin | Q86YZ3 | ENSG00000197915 | HPRR4050552 | LLMIFKLVQAHNKIIGKDYCQVSGSKLRDDTHQHQEEQEETEKEENKRQES | 51 | Literature[2] |
| HSPA4 | heat shock protein family A (Hsp70) member 4 | P34932 | ENSG00000170606 | HPRR1920102 | FEELGKQIQQYMKIISSFKNKEDQYDHLDAADMTKVEKSTNEAMEWMNNKLNLQNKQSLTMDPVVKSKEIEAKIKELTSTCSPIISKPKPKVEPPKEEQKNAEQNGPVDG | 110 | mRNA screening[4] |
| HSPA4 | heat shock protein family A (Hsp70) member 4 | P34932 | ENSG00000170606 | HPRR4220528 | QEEPHVEEQQQQTPAENKAESEEMETSQAGSKDKKMDQPPQAKKAKVKTSTV | 52 | mRNA screening[4] |
| IFITM2;IFITM3 | interferon induced transmembrane protein 2; interferon induced transmembrane protein 3 | Q01629; Q01628 | ENSG00000185201; ENSG00000142089 | HPRR370324 | MNHTVQTFFSPVNSGQPPNYEMLKEEHEVAVLGAPHNPAPPTSTVIHIRSETSVPDH | 57 | mRNA screening[4] |
| LCN2 | lipocalin 2 | P80188 | ENSG00000148346 | HPRR400092 | QDSTSDLIPAPPLSKVPLQQNFQDNQFQGKWYVVGLAGNAILREDKDPQKMYATIYELKEDKSYNVTSVLFRKKKCDYWIRTFVPGCQPGEFTLGNIKSYPGLTSYLVRVVSTNYNQHAMVFFKKVSQNREYFKITL | 137 | mRNA screening[4] |
| LGALS1 | galectin 1 | P09382 | ENSG00000100097 | HPRR220971 | EVAPDAKSFVLNLGKDSNNLCLHFNPRFNAHGDANTIVCNSKDGGAWGTEQREAVFPFQPGSVAEVCITFDQANLTVKLPDGYEFKFPNRLNLEAINYMAADGDFKIKCV | 110 | mRNA screening[4] |
| LGALS1 | galectin 1 | P09382 | ENSG00000100097 | HPRR252469 | LKPGECLRVRGEVAPDAKSFVLNLGKDSNNLCLHFNPRFNAHGDANTIVCNSKDGGAWGTEQREAVFPFQPGSVAEVCITFDQANLTVKLPDGYEFKFPNRLNLEAINYMAADGDF | 116 | mRNA screening[4] |
| LTF | lactotransferrin | P02788 | ENSG00000012223 | HPRR3720081 | PNHAVVSRMDKVERLKQVLLHQQAKFGRNGSDCPDKFCLFQSETKNLLFNDNTECLARLHGK | 62 | mRNA screening[4] |
| LTF | lactotransferrin | P02788 | ENSG00000012223 | HPRR3720082 | RVVWCAVGEQELRKCNQWSGLSEGSVTCSSASTTEDC | 37 | mRNA screening[4] |
| LYZ | lysozyme | P61626 | ENSG00000090382 | HPRR3700032 | WCNDGKTPGAVNACHLSCSALLQDNIADAVACAKRVVRDPQGIRAWVAWRNRCQNRDVRQYVQGCG | 66 | mRNA screening[4] |
| LYZ | lysozyme | P61626 | ENSG00000090382 | HPRR3700033 | GKVFERCELARTLKRLGMDGYRGISLANWMCLAKWESGYNTRATNYNAGDRSTDY | 55 | mRNA screening[4] |
| MAN1A1 | mannosidase alpha class 1A member 1 | P33908 | ENSG00000111885 | HPRR3700097 | MKHEFEEAKSWVEENLDFNVNAEISVFEV | 29 | DNA methylation screening[4] |
| MAN1A1 | mannosidase alpha class 1A member 1 | P33908 | ENSG00000111885 | HPRR3700098 | TLQKLPEEIQRDILLEKKKVAQDQLRDKAPFRGLPPVDFVPPIGVESREPADAAIREK | 58 | DNA methylation screening[4] |
| MED1 | mediator complex subunit 1 | Q15648 | ENSG00000125686 | HPRR3830164 | SHPVDNKWTPSFSSITSANSVDLPACFFLKFPQPIPVSRAFVQKLQNCTGIPLFETQPTYAPLYELITQFELSKDPDPIPLNHNMRFYAALPGQQH | 96 | DNA methylation screening[4] |
| MED1 | mediator complex subunit 1 | Q15648 | ENSG00000125686 | HPRR3830165 | MEKRVVMSSGGHQHLVSCLETLQKALKVTSLPAMTDRLESIARQNGLGSHLSASGTECYITSDMFYVEVQLDPAGQLCDVKVAHHGENPVSCP | 93 | DNA methylation screening[4] |
| MED13L | mediator complex subunit 13 like | Q71F56 | ENSG00000123066 | HPRR3070311 | PALYGNGLELQQLSTLDDRTVLVGQRLPLMAEVSETALYCGIRPSNPESSEKWWHSYRLPPSDDAEFRPPELQGERCDAKMEVNSESTALQRLLAQPNKRFKIWQD | 106 | DNA methylation screening[4] |
| MED13L | mediator complex subunit 13 like | Q71F56 | ENSG00000123066 | HPRR3070312 | MMCQSTFLPQVEGTKKPQEPPISLLLLLQNQHTQPFASLNFLDYISSNNRQTLPCVSWSYDRVQADNNDYWTECFNALE | 79 | DNA methylation screening[4] |
| MNDA | myeloid cell nuclear differentiation antigen | P41218 | ENSG00000163563 | HPRR4190869 | EKSKVAKKIKTQEKAPVKKINQEEVGLAAPAPTARNKLTSEARGRIPVAQKRKTPNKEKTEAKRNKVSQEQSKPPGPSGASTSAAVDHP | 89 | mRNA screening[4] |
| MNDA | myeloid cell nuclear differentiation antigen | P41218 | ENSG00000163563 | HPRR610035 | PQTSSSTPSNTSFTPNQETQAQRQVDARRNVPQNDPVTVVVLKATAPFKYESPENGKSTMFHATVASKTQYFHVKVFDINLKEKFVRKKVITISDYSECKGVMEIKEASSVSDFN | 115 | mRNA screening[4] |
| NCF4 | neutrophil cytosolic factor 4 | Q15080 | ENSG00000100365 | HPRR2800094 | MAVAQQLRAESDFEQLPDDVAISANIADIEEKRGFTSHFVFVIEVKTKGGSKYLIYRRY | 59 | mRNA screening[4] |
| NCF4 | neutrophil cytosolic factor 4 | Q15080 | ENSG00000100365 | HPRR2800095 | EDIALNYRDAEGDLVRLLSDEDVALMVRQARGLPSQKRLFPWKLHITQKDNYRVYNTMP | 59 | mRNA screening[4] |
| NCOR2 | nuclear receptor corepressor 2 | Q9Y618 | ENSG00000196498 | HPRR430039 | EEPVKSECTEEAEEGPAKGKDAEAAEATAEGALKAEKKEGGSGRATTAKSSGAPQDSDSSATCSADEVDEAEGGDKNRLLSPRPSLLTPTGDPRANASPQKPLDLKQLKQRAAAIPPIQVTKV | 123 | DNA methylation screening[4] |
| PADI1 | peptidyl arginine deiminase 1 | Q9ULC6 | ENSG00000142623 | HPRR2550898 | PKRVVQLSLKMPTHAVCVVGVEAHVDIHSDVPKGANSFRVSGSSGVEVFMVYNRTRVKEPIGKARWPLDT | 70 | Literature[2] |
| PADI1 | peptidyl arginine deiminase 1 | Q9ULC6 | ENSG00000142623 | HPRR3930191 | PSACLKLFQEKKEEGYGEAAQFDGLKHQAKRSINEMLADRHLQRDNLHAQKCIDWN | 56 | Literature[2] |
| PADI2 | peptidyl arginine deiminase 2 | Q9Y2J8 | ENSG00000117115 | HPRR3700197 | PWLPKEDCRDEKVYSKEDLKDMSQMILRTKGPDRLPAGYEIVLYISMSDSDKVGVFYVENPFFGQRYIHILGRRKLYHVVKYTGGS | 86 | Literature[2] |
| PADI3 | peptidyl arginine deiminase 3 | Q9ULW8 | ENSG00000142619 | HPRR3500166 | FRMLLASPGACFKLFQEKQKCGHGRALLFQGVVDDEQVKTISINQVLSNKDLINYNKFVQSCIDWNREV | 69 | Literature[2] |
| PADI4 | peptidyl arginine deiminase 4 | Q9UM07 | ENSG00000159339 | HPRR1060019 | GTLTQLDICSSAPEDCTSFSINASPGVVVDIAHGPPAKKKSTGSSTWPLDPGVEVTLTMKVASGSTGDQKVQISYYGPKTPPVKALLYLTGV | 92 | Literature[2] |
| PADI4 | peptidyl arginine deiminase 4 | Q9UM07 | ENSG00000159339 | HPRR1060020 | CEDDEVLDSEDLQDMSLMTLSTKTPKDFFTNHTLVLHVARSEMDKVRVFQATRGKLSSKCSVVLGPKWPSHYLMVPGGKHNMDFYVEALAFPDTDFPGLITLTISLLD | 108 | Literature[2] |
| PADI6 | peptidyl arginine deiminase 6 | Q6TGC4 | ENSG00000276747 | HPRR2552151 | SACYKLFREKQKEGYGDALLFDELRADQLLSNGREAKTIDQLLADESLKKQNEYVEKCIHLNRDILKTELGLVEQDIIEIPQLFCLEKLTNIPSDQQPK | 99 | Literature[2] |
| PADI6 | peptidyl arginine deiminase 6 | Q6TGC4 | ENSG00000276747 | HPRR2552153 | ETFYVEAIAFPSAEFSGLISYSVSLVEESQDPSIPETVLYKDTVVFRVAPCVFIPCTQVPLEVYLCRELQLQGFVDTVTKLSEK | 84 | Literature[2] |
| PRDX1 | peroxiredoxin 1 | Q06830 | ENSG00000117450 | HPRR1370091 | KGKYVVFFFYPLDFTFVCPTEIIAFSDRAEEFKKLNCQVIGASVDSHFCHLAWVNTPKKQGGLGPMNIPLVSDPKRTIAQDYGVLKADEGISFRGLFIIDDKGILRQITVNDLPVGRSVDETLRL | 125 | mRNA screening[4] |
| RPTN | repetin | Q6XPR3 | ENSG00000215853 | HPRR2551740 | SSHYIQSQTGEIQGQNKYFQGTEGTRKASYVEQSGRSGRLSQQTPGQEGYQNQGQGFQSRDSQQNGHQVWEPEEDSQHHQHKLLAQIQQERP | 92 | Literature[2] |
| RPTN | repetin | Q6XPR3 | ENSG00000215853 | HPRR2551741 | ETVETILNLLDQDRDGHIDFHEYLLLVFQLVQACYHKLDNKSHGGRTSQQERGQEGAQDCKFPGNTGRQHRQRHEEERQNSHHSQ | 85 | Literature[2] |
| RPTN | repetin | Q6XPR3 | ENSG00000215853 | HPRR2551743 | KSGSYCGQSERLGQELGCGQTDRQGQSSHYGQTDRQDQSYHYGQTDRQGQSSHYSQTDRQGQSSHYSQPDRQGQSSHYGQMD | 82 | Literature[2] |
| S100A1 | S100 calcium binding protein A1 | P23297 | ENSG00000160678 | HPRR1420141 | GSELETAMETLINVFHAHSGKEGDKYKLSKKELKELLQTELSGFLDAQKDVDAVDKVMKELDENGDGEVDFQEYVVLVAALTVACNNFFWENS | 93 | Literature[2] |
| S100A2 | S100 calcium binding protein A2 | P29034 | ENSG00000196754 | HPRR2760377 | KELLHKELPSFVGEKVDEEGLKKLMGSLDENSDQQVDFQEYAVFLALITVMCNDFFQGCP | 60 | Literature[2] |
| S100A2 | S100 calcium binding protein A2 | P29034 | ENSG00000196754 | HPRR4090188 | EMKELLHKELPSFVGEKVDEEGLKKLMGSLDENSDQQVD | 39 | Literature[2] |
| S100A4 | S100 calcium binding protein A4 | P26447 | ENSG00000196154 | HPRR1350146 | MACPLEKALDVMVSTFHKYSGKEGDKFKLNKSELKELLTRELPSFLGKRTDEAAFQKLMSNLDSNRDNEVDFQEYCVFLSCIAMMCNEFFEGFPDKQPRKK | 101 | Literature[2] |
| S100A5 | S100 calcium binding protein A5 | P33763 | ENSG00000196420 | HPRR2552087 | GREGSKLTLSRKELKELIKKELCLGEMKESSIDDLMKSLDKNSDQEIDFKEYSVFLTMLCMAYNDFFLEDNK | 72 | Literature[2] |
| S100A5 | S100 calcium binding protein A5 | P33763 | ENSG00000196420 | HPRR3790623 | KELIKKELCLGEMKESSIDDLMKSLDKNSDQEIDFKEYSVFLTMLCMAYNDFFLEDNK | 58 | Literature[2] |
| S100A6 | S100 calcium binding protein A6 | P06703 | ENSG00000197956 | HPRR1420196 | AIFHKYSGREGDKHTLSKKELKELIQKELTIGSKLQDAEIARLMEDLDRNKDQEVNFQEYVTFLGALALIYNE | 73 | mRNA screening[4], Literature[2] |
| S100A7A;S100A7 | S100 calcium binding protein A7A; S100 calcium binding protein A7 | Q86SG5; P31151 | ENSG00000184330; ENSG00000143556 | HPRR1420206 | EKPSLLTMMKENFPNFLSACDKKGTNYLADVFEKKDKNEDKKIDFSEFLSLLGDIATDYHKQSHGAAPCS | 70 | Literature[2] |
| S100A7L2 | S100 calcium binding protein A7 like 2 | Q5SY68 | ENSG00000197364 | HPRR3420742 | SDMDYLSNALEKKDDNKDKKVNYSE | 25 | Literature[2] |
| S100A9 | S100 calcium binding protein A9 | P06702 | ENSG00000163220 | HPRR400189 | CKMSQLERNIETIINTFHQYSVKLGHPDTLNQGEFKELVRKDLQNFLKKENKNEKVIEHIMEDLDTNADKQLSFEEFIMLMARLTWASHEKMHEGDEG | 98 | mRNA screening[4], Literature[2] |
| S100A10 | S100 calcium binding protein A10 | P60903 | ENSG00000197747 | HPRR410031 | MPSQMEHAMETMMFTFHKFAGDKGYLTKEDLRVLMEKEFPGFLENQKDPLAVDKIMKDLDQCRDGKV | 67 | Literature[2] |
| S100A11 | S100 calcium binding protein A11 | P31949 | ENSG00000163191 | HPRR3410200 | SLIAVFQKYAGKDGYNYTLSKTEFLSFMNTELAAFTKNQKDPGVLDRMMKKLDTNSDGQLDFSEFLNLIGGLAMACHDSF | 80 | Literature[2] |
| S100A12 | S100 calcium binding protein A12 | P80511 | ENSG00000163221 | HPRR400165 | KLEEHLEGIVNIFHQYSVRKGHFDTLSKGELKQLLTKELANTIKNIKDKAVIDEIFQGLDANQDEQVDFQEFISLVAIALKAAHYHTHKE | 90 | mRNA screening[4], Literature[2] |
| S100A13 | S100 calcium binding protein A13 | Q99584 | ENSG00000189171 | HPRR2330156 | ELEESIETVVTTFFTFARQEGRKDSLSVNEFKELVTQQLPHLLKDVGSLDEKMKSLDVNQDSELKFNEYWRLIGELAKEIRKKKDLKI | 88 | Literature[2] |
| S100A14 | S100 calcium binding protein A14 | Q9HCY8 | ENSG00000189334 | HPRR2552068 | GQCRSANAEDAQEFSDVERAIETLIKNFHQYSVEGGKETLTPSELRDLVTQQLPHLMPSNCGLEEKIANLGSCNDSKLEFRSFWELIGEA | 90 | Literature[2] |
| S100A16 | S100 calcium binding protein A16 | Q96FQ6 | ENSG00000188643 | HPRR2552038 | VIVLVENFYKYVSKYSLVKNKISKSSFREMLQKELNHMLSDTGNRKAADKLIQNLDANHDGRISFDEYWTLIGGITGPIAKLIHEQEQ | 88 | Literature[2] |
| S100G | S100 calcium binding protein G | P29377 | ENSG00000169906 | HPRR233077 | KSPEELKRIFEKYAAKEGDPDQLSKDELKLLIQAEFPSLLKGPNTLDDLFQELDKNGDGEVSFE | 64 | Literature[2] |
| S100G | S100 calcium binding protein G | P29377 | ENSG00000169906 | HPRR3920159 | KSPEELKRIFEKYAAKEGDPDQLSKDELKLLIQAEFPSLLKGPNTLDDLFQELDK | 55 | Literature[2] |
| S100P | S100 calcium binding protein P | P25815 | ENSG00000163993 | HPRR2350024 | MTELETAMGMIIDVFSRYSGSEGSTQTLTKGELKVLMEKELPGFLQSGKDKDAVDKLLKDLDANGDAQVDFSEF | 74 | mRNA screening[4], Literature[2] |
| S100P | S100 calcium binding protein P | P25815 | ENSG00000163993 | HPRR3970245 | FIVFVAAITSACHKYFEKAGL | 21 | mRNA screening[4], Literature[2] |
| SPRR3 | small proline rich protein 3 | Q9UBC9 | ENSG00000163209 | HPRR1420163 | EPCHSKVPQPGNTKIPEPGCTKVPEPGCTKVPEPGCTKVPEPGCTKVPEPGCTKVPEPGYTKVPEPGSIKVPDQGFIKFPEPGAIKVPEQGYTKVPVP | 98 | Literature[3] |
| SRGN | serglycin | P10124 | ENSG00000122862 | HPRR252377 | MQKLLKCSRLVLALALILVLESSVQGYPTRRARYQWVRCNPDSNSANCLEEKGPMFELLPGESNKIPRLRTDLFPKTRIQDLNRIFPLSEDYSGSGFGSGSGSGSGSGSGFLTEMEQDYQLVDE | 124 | mRNA screening[4] |
| TCHH | trichohyalin | Q07283 | ENSG00000159450 | HPRR2551234 | QEKSRREEQELWQEEEQKRRQERERKLREEHIRRQQKEEQRHRQVGEIKSQEGKGHGRLLEPGTHQFASVPVRSSPLYEY | 80 | Literature[2] |
| TCHHL1 | trichohyalin like 1 | Q5QJ38 | ENSG00000182898 | HPRR3410198 | KHSNIQEPPLQREDEPSSQHADLPEQAAARSPSQTQKSTDSKDVCRMFDTQEPGKDADQTPAKTKNLGEPEDYGRTSETQEKECETKDLPVQYGSRN | 97 | Literature[2] |
| TCHHL1 | trichohyalin like 1 | Q5QJ38 | ENSG00000182898 | HPRR3410199 | RDQEPCSVERGAVYSSPLYQYLQEKILQQTNVTQEEHQKQVQIAQASGPELCSVSLTSEISDCSVFFNYSQASQPYTRGLPLDESPAGAQETPAPQ | 96 | Literature[2] |
| TGM1 | transglutaminase 1 | P22735 | ENSG00000092295 | HPRR3140167 | DLSLTLLGAAVVGQECEVQIVFKNPLPVTLTNVVFRLEGSGLQRPKILNVGDIGGNETVTLRQSFVPVRPGPRQLIASLD | 80 | Literature[2] |
| TGM1 | transglutaminase 1 | P22735 | ENSG00000092295 | HPRR3140168 | MMDGPRSDVGRWGGNPLQPPTTPSPEPEPEPDGRSRRGGGRSFWARCCGCCSCRNAADDDWGPEPSDSRGRGSSSGTRRPGSRGSDSRRPVSRGSGVNAA | 100 | Literature[2] |
| TGM1 | transglutaminase 1 | P22735 | ENSG00000092295 | HPRR3140170 | NSDKVYWQRQDDGSFKIVYVEEKAIGTLIVTKAISSNMREDITYLYKHPEGSDAERKAVETAAAHGSKPNVYANRGSAEDVAMQV | 85 | Literature[2] |
| TGM2 | transglutaminase 2 | P21980 | ENSG00000198959 | HPRR2430028 | MAEELVLERCDLELETNGRDHHTADLCREKLVVRRGQPFWLTLHFEGRNYEASVDSLTFSVVTGPAPSQEAGTK | 74 | Literature[2] |
| TGM2 | transglutaminase 2 | P21980 | ENSG00000198959 | HPRR2430030 | VEPVINSYLLAERDLYLENPEIKIRILGEPKQKRKLVAEVSLQNPLPVALEGCTFTVEGAGLTEEQKTVEIPDPVEAGEEVKVRMDLLP | 89 | Literature[2] |
| TGM3 | transglutaminase 3 | Q08188 | ENSG00000125780 | HPRR4320672 | MAALGVQSINWQKAFNRQAHHTDKFSSQELILRRGQNFQVLMIMNKGLGSNERLEFIVSTGPYPSESAMTKAVFPLSNGSSGGWSAVLQASN | 92 | Literature[2] |
| TGM4 | transglutaminase 4 | P49221 | ENSG00000163810 | HPRR2700176 | SVNFTVILKRKTAALQNVNILGSFELQLYTGKKMAKLCDLNKTSQIQGQVSEVTLTLDSKTYINSLAILDDEPVIRGFIIAEIVESKEIMA | 91 | Literature[2] |
| TGM4 | transglutaminase 4 | P49221 | ENSG00000163810 | HPRR2700177 | YPEFSIELPNTGRIGQLLVCNCIFKNTLAIPLTDVKFSLESLGISSLQTSDHGTVQPGETIQSQIKCTPIKTGPKKFIVKLSSKQVK | 87 | Literature[2] |
| TGM5 | transglutaminase 5 | O43548 | ENSG00000104055 | HPRR3100089 | FLKALQKLKARSFHGSQRGAELQPSRPTSLSQDSPRSLHTPSLRPSDVVQVSLKFKLLDPPNMGQDICFVLLALNMSSQFKDLKVNLSAQSLLHD | 95 | Literature[2] |
| TGM5 | transglutaminase 5 | O43548 | ENSG00000104055 | HPRR3100091 | VRAIKEGEVDLNYDTPFVFSMVNADCMSWLVQGGKEQKLHQDTSSVGNFISTKSIQSDERDDITENYKY | 69 | Literature[2] |
| TGM6 | transglutaminase 6 | O95932 | ENSG00000166948 | HPRR3310212 | GSRKERQVYSKAVNRLFGVEASGRRIWIRRAGGRCLWRDDLLEPATKPSIAGKFKVLEPPMLGHDLRLALCLANLTSRAQRVRVNLSGATILYTR | 95 | Literature[2] |
| TGM7 | transglutaminase 7 | Q96PF1 | ENSG00000159495 | HPRR2700160 | VTLTNTLMVALSSCTMVLEGSGLINGQIAKDLGTLVAGHTLQIQLDLYPTKAGPRQLQVLISSNEVKEIKGYKDIFVTVAGA | 82 | Literature[2] |
| TOX2 | TOX high mobility group box family member 2 | Q96NM4 | ENSG00000124191 | HPRR3300261 | IAGVFPQKFDGDSAYVGMSDGNPELLSTSQTYNGQSENNEDYEIPPITPPN | 51 | DNA methylation screening[4] |
| TOX2 | TOX high mobility group box family member 2 | Q96NM4 | ENSG00000124191 | HPRR3300264 | HEASYHSLCHGLTPNGLLPAYSYQAMDLPAIMVSNMLAQDSHLLSGQLPTIQEMVHSEVAAYDSGR | 66 | DNA methylation screening[4] |

^1^Cuppari C, Manti S, Salpietro A, Valenti S, Capizzi A, Arrigo T, et al. HMGB1 levels in children with atopic eczema/dermatitis syndrome (AEDS). Pediatr Allergy Immunol. 2016;27:99-102.

^2^Kizawa K, Takahara H, Unno M, Heizmann CW. S100 and S100 fused-type protein families in epidermal maturation with special focus on S100A3 in mammalian hair cuticles. Biochimie. 2011;93:2038-47.

^3^Marenholz I, Rivera VA, Esparza-Gordillo J, Bauerfeind A, Lee-Kirsch MA, Ciechanowicz A, et al. Association screening in the Epidermal Differentiation Complex (EDC) identifies an SPRR3 repeat number variant as a risk factor for eczema. J Invest Dermatol. 2011;131:1644-9.

^4^Katayama S, Bruhn S, Scheynius A, Lundeberg L, Kere J, Andersson A, et al. Epigenetic modifications in skin-homing CD4+CLA+ T-cells of atopic dermatitis patients relate to mRNA expression changes in high mobility group proteins. at Conference: 2(nd) Inflammatory Skin Disease Summit, November 16-19, 2016, New York. Exp Dermatol. 2016;25 Suppl 4:36-7.

**Table S2.** Characterization of the AD patients and healthy controls subdivided according to IgG reactivity^d)^

|  | | **N** | **Gender**  **F / M**  **n**  **(%F)** | **Age (years)**  **median**  **(range)** | **SCORAD^a)^**  **median**  **(range)** | **Rhino-conjunctivitis and/or asthma**  **past or present**  **n**  **(%)** | **Total plasma IgE^b)^**  **≥122 kU/L**  **n**  **(%)** | | **Total**  **plasma IgE^b)^ (kU/L)**  **median (range)** | **Phadiatop positive ^c)^**  **n**  **(%)** | |
| --- | --- | --- | --- | --- | --- | --- | --- | --- | --- | --- | --- |
| AD patients  with IgG- reactivity to **KRTAP17-1** | | 55 | 30 / 25  (55) | 28  (18 – 63) | 35  (17 – 66) | 43  (78) | 28  (51) | 130  (4.5 - 12835) | | | 42  (76) |
| AD patients  with IgG-reactivity to **HSPA4** | | 36 | 18 / 18  (50) | 25  (18 – 62) | 36  (16 – 69) | 28  (78) | 19  (53) | 140  (12 - 5150) | | | 27  (75) |
| AD patients  with IgG-reactivity to **S100A12** | | 13 | 6 / 7  (46) | 22  (18 – 57) | 36  (12 – 65) | 9  (69) | 4  (31) | 90  (12 - 1100) | | | 7  (54) |
| AD patients  with IgG-reactivity to **S100Z** | | 8 | 2 / 6  (25) | 42  (18 – 65) | 49  (19 – 67) | 8  (100) | 4  (50) | 330  (13 - 9800) | | | 8  (100) |
| Severe^a)^  with IgG-reactivity to **KRTAP17-1** | 20 | 6 / 14  (30) | 28  (18 - 54) | 51  (42 - 66) | 16  (80) | 14  (70) | 390  (4.5 - 12835) | | | 17  (85) |  |
| Severe^a)^  with IgG-reactivity to **HSPA4** | 15 | 4 / 11  (27) | 24  (18 - 57) | 51  (42 - 69) | 13  (87) | 11  (73) | 490  (45 - 5150) | | | 12  (80) |  |
| Severe^a)^  with IgG-reactivity to **S100A12** | 5 | 2 / 3 (40) | 22  (18 - 27) | 51  (44 - 65) | 4  (80) | 3  (60) | 200  (45 - 1100) | | | 3  (60) |  |
| Severe^a)^  with IgG-reactivity to **S100Z** | 7 | 1 / 6 (14) | 42  (18 – 65) | 53  (41 - 67) | 7  (100) | 3  (43) | 89  (13 - 9800) | | | 7  (100) |  |
| Moderate^a)^  with IgG-reactivity to K**RTAP17-1** | 35 | 24 / 11  (69) | 29  (18 - 63) | 31  (17 – 40) | 27  (77) | 14  (40) | 89  (16 – 6810) | | | 25  (71) |  |
| Moderate^a)^  with IgG-reactivity to **HSPA4** | 21 | 14 / 7  (67) | 25  (18 - 62) | 27  (16 – 40) | 15  (71) | 8  (38) | 100  (12 – 2800) | | | 15  (71) |  |
| Moderate^a)^  with IgG-reactivity to **S100A12** | 8 | 4 / 4  (50) | 27  (18 - 57) | 30  (19 – 40) | 5  (62) | 1  (12) | 76  (12 – 240) | | | 4  (50) |  |
| Moderate^a)^  with IgG-reactivity to **S100Z** | 1 | 1 / 0  (100) | 30  (30) | 19  (19) | 1  (100) | 1  (100) | 570  (570) | | | 1  (100) |  |
| Healthy controls  with IgG- reactivity to **KRTAP17-1** | | 13 | 8 / 5  (62) | 35  (20 – 62) | NA | 0  (0) | 0  (0) | 17  (3.2 – 70) | | | 0  (0) |
| Healthy controls  with IgG-reactivity to **HSPA4** | | 9 | 5 / 4  (56) | 38  (22 – 61) | NA | 0  (0) | 0  (0) | 17  (2.4 – 66) | | | 0  (0) |
| Healthy controls  with IgG-reactivity to **S100A12** | | 0 | NA | NA | NA | NA | NA | NA | | | NA |
| Healthy controls  with IgG-reactivity to **S100Z** | | 3 | 1 / 2  (33) | 24  (23 – 42) | NA | 0 | 0 | 17  (11 – 35) | | | 0 |

a) Objective SCORAD^[5]^, severe AD defined as SCORAD ≥41.

b) ImmunoCAP (Thermo Fisher Scientific), reference range 1.6 – 122 kU/L.

c) Phadiatop (Thermo Fisher Scientific), plasma IgE to any of 11 common aeroallergens, positive ≥ 0.35 kU/L.

d) IgG reactivity were defined using sample-specific cutoffs based on the sample median across protein fragments and a factor times the median absolute deviation (MAD), see Methods section.

AD = atopic dermatitis, F = Female, M = Male, N = number of individuals, n = number of positive individuals, NA = not applicable.

**Table S3:** IgG-reactivity^a)^ and adjusted MFI^b)^ to four antigens detected in the targeted screening of the complete cohort using antigen bead arrays subdivided by Phadiatop and total plasma IgE

| **Gene** | | **KRTAP17-1** | **HSPA4** | **S100A12** | **S100Z** |
| --- | --- | --- | --- | --- | --- |
| **Number of IgG-reactive individuals** | | n (%) | n (%) | n (%) | n (%) |
| AD patients (N=173) | | 55 (32) | 36 (21) | 13 (8) | 8 (5) |
|  | Phadiatop positive^c)^ (N=132) | 42 (32) | 27 (20) | 7 (5) | 8 (6) |
|  | Phadiatop negative^c)^ (N=41) | 13 (32) | 9 (22) | 6 (15) | 0 (0) |
|  | Elevated total IgE^d)^ (N=95) | 28 (29) | 19 (20) | 4 (4) | 4 (4) |
|  | Normal total IgE^d)^ (N=78) | 27 (35) | 17 (22) | 9 (12) | 4 (5) |
| Healthy Controls^e)^ (N=84) | | 13 (15) | 9 (11) | 0 (0) | 3 (4) |
| ***P* values**^f)^  **Calculated on proportion positive individuals** | |  |  |  |  |
| AD Phadiatop^positive^ vs AD Phadiatop^negative^ | | 1.00 | 0.83 | 0.08 | 0.20 |
| AD Phadiatop^positive^ vs HC | | 0.01 | 0.06 | 0.04 | 0.53 |
| AD Phadiatop^negative^ vs HC | | 0.06 | 0.11 | 0.001 | 0.55 |
| AD Total IgE^high^ vs AD Total IgE^normal^ | | 0.51 | 0.85 | 0.09 | 1.00 |
| AD Total IgE^high^ vs HC | | 0.03 | 0.10 | 0.12 | 1.00 |
| AD Total IgE^normal^ vs HC | | 0.006 | 0.09 | 0.001 | 0.71 |
| **Adjusted MFI of individuals** | | median (Q1 - Q3) | median (Q1 - Q3) | median (Q1 - Q3) | median (Q1 - Q3) |
| AD Phadiatop^positive^ | | 7.2 (4.3 - 18.9) | 3.2 (1.3 - 9.8) | 2.2 (0.8 - 5.7) | -1.7 (-2.1 - -0.6) |
| AD Phadiatop^negative^ | | 7.2 (4.8 - 16.9) | 3.4 (1.3 - 9.8) | 4.2 (0.8 - 7.0) | -1.8 (-2.2 - -0.5) |
| AD Total IgE^high^ | | 7.0 (4.3 - 16.5) | 3.2 (1.4 - 8.7) | 2.4 (1.0 - 5.8) | -1.7 (-2.1 - -0.6) |
| AD Total IgE^normal^ | | 7.8 (4.7 - 21.0) | 3.3 (1.2 - 9.9) | 2.7 (0.7 - 6.9) | -1.7 (-2.1 - -0.5) |
| HC | | 5.8 (3.4 - 10.5) | 2.8 (1.1 - 7.2) | 0.8 (0.2 - 2.0) | -1.6 (-2.0 - -0.2) |
| ***P* values**^g)^  **Calculated on adjusted MFI values** | |  |  |  |  |
| AD Phadiatop^positive^ vs AD Phadiatop^negative^ | | 0.93 | 0.81 | 0.32 | 0.43 |
| AD Phadiatop^positive^ vs HC | | 0.02 | 0.40 | 0.00002 | 0.30 |
| AD Phadiatop^negative^ vs HC | | 0.06 | 0.46 | 0.0002 | 0.09 |
| AD Total IgE^high^ vs AD Total IgE^normal^ | | 0.47 | 0.94 | 0.71 | 0.96 |
| AD Total IgE^high^ vs HC | | 0.06 | 0.42 | 0.00002 | 0.25 |
| AD Total IgE^normal^ vs HC | | 0.01 | 0.43 | 0.0001 | 0.20 |

a) IgG reactivity were defined using sample-specific cutoffs based on the sample median across protein fragments and a factor times the median absolute deviation (MAD), see Methods.

b) Sample median across protein fragments was subtracted from each signal and divided by the MAD, see Methods.

c) Phadiatop (Thermo Fisher Scientific), plasma IgE-reactivity to any of 11 common aeroallergens, reference range ≥ 0.35 kU/L.

d) ImmunoCAP (Thermo Fisher Scientific), reference range 1.6 – 122 kU/L.

e) All HC were negative in Phadiatop and had normal total IgE levels.

f) Fisher's exact test.

g) Wilcoxon rank-sum test.

AD = atopic dermatitis, HC = healthy controls, N= number of individuals, n = number of positive individuals


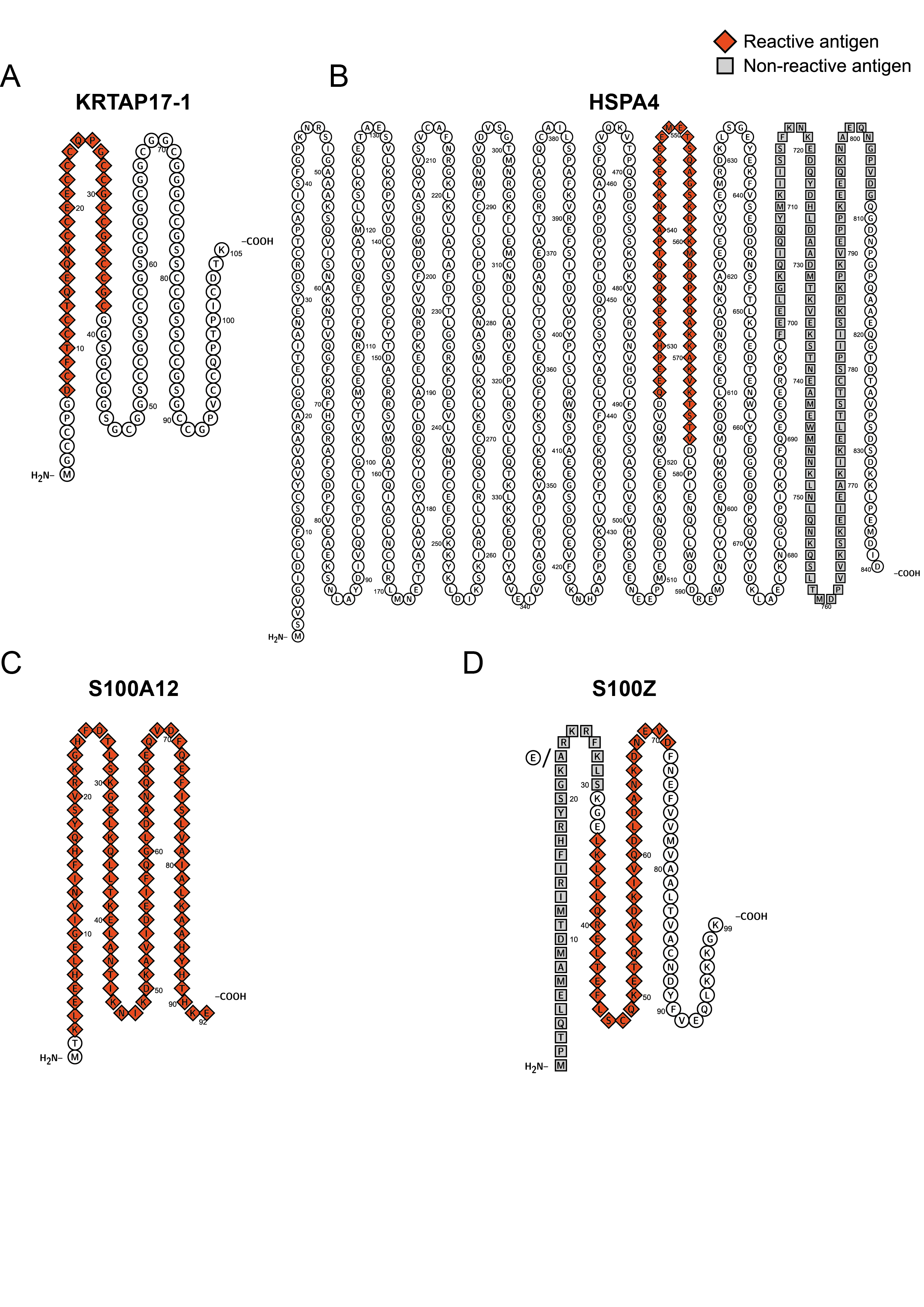


**Fig S1. Protein sequence and antigen regions.** Illustration of the complete protein sequence of (**A**) KRTAP17-1, (**B**) HSPA4, (**C**) S100A12, and (**D**) S100Z. The protein sequence is here shown with the one letter abbreviation for each amino acid in white circles, red diamonds or grey squares. The regions of the proteins that were represented as protein fragments and that were identified as either reactive or non-reactive in the targeted analysis are highlighted in red (diamonds, reactive) and grey (squares, non-reactive).

# References

1. Cuppari C, Manti S, Salpietro A, Valenti S, Capizzi A, Arrigo T, et al. HMGB1 levels in children with atopic eczema/dermatitis syndrome (AEDS). Pediatr Allergy Immunol. 2016;27(1):99-102.

2. Kizawa K, Takahara H, Unno M, Heizmann CW. S100 and S100 fused-type protein families in epidermal maturation with special focus on S100A3 in mammalian hair cuticles. Biochimie. 2011;93(12):2038-47.

3. Marenholz I, Rivera VA, Esparza-Gordillo J, Bauerfeind A, Lee-Kirsch MA, Ciechanowicz A, et al. Association screening in the Epidermal Differentiation Complex (EDC) identifies an SPRR3 repeat number variant as a risk factor for eczema. J Invest Dermatol. 2011;131(8):1644-9.

4. Katayama S, Bruhn S, Scheynius A, Lundeberg L, Kere J, Andersson A, et al. Epigenetic modifications in skin-homing CD4+CLA+ T-cells of atopic dermatitis patients relate to mRNA expression changes in high mobility group proteins. at Conference: 2(nd) Inflammatory Skin Disease Summit, November 16-19, 2016, New York. Exp Dermatol. 2016;25 Suppl 4:36-7.

5. Kunz B, Oranje AP, Labreze L, Stalder JF, Ring J, Taieb A. Clinical validation and guidelines for the SCORAD index: consensus report of the European Task Force on Atopic Dermatitis. Dermatology. 1997;195(1):10-9.
